# Accelerating Electrochemical Responses of Na_4_VMn(PO_4_)_3_ via Bulk‐Defects and Architecture Engineering for High‐Performance Sodium‐Ion Batteries

**DOI:** 10.1002/advs.202415331

**Published:** 2025-04-17

**Authors:** Jingwen Zhao, Bo Zou, Weitao Yan, Shijia Li, Wentao Wu, Wei‐Hua Wang, Shiyu Li, Ying Bai

**Affiliations:** ^1^ Key Laboratory for High‐Efficiency Energy Conversion Science and Technology of Henan Province International Joint Research Laboratory of New Energy Materials and Devices of Henan Province School of Physics and Electronics Henan University Kaifeng 475004 P. R. China; ^2^ School of Science and Ministry of Industry and Information Technology Key Laboratory of Micro‐Nano Optoelectronic Information System Harbin Institute of Technology Shenzhen 518055 P. R. China; ^3^ Department of Micro/Nano Electronics Tianjin Key Laboratory of Photo‐Electronic Thin Film Device and Technology Engineering Research Center of Thin Film Optoelectronics Technology (Ministry of Education) Nankai University Tianjin 300350 P. R. China

**Keywords:** bulk‐defects and architecture engineering, high‐performance, NASICON, sodium‐ion batteries

## Abstract

Manganese‐based NASICON‐type Na_4_VMn(PO_4_)_3_ (NVMP) has captured widespread attention in sodium‐ion batteries (SIBs) due to its abundant reserves and high operating voltages. However, the low intrinsic conductivity and detrimental Jahn–teller (J–T) effect impedes its electron and ion transfer, leading to rapid structural degradation and capacity decay. Herein, a facile multiscale coupling strategy is proposed to synthesize the nanosheet‐stacked rods (NVMP‐NSRs) with rational defects for improving intrinsic conductivity and structural stability, thus accelerating electrochemical responses. Localized unsaturated coordination states around vanadium atoms in NVMP‐NSRs are also regulated, further facilitating rapid Na^+^ diffusion with relieved volume expansion due to the unique architecture design. Density functional theory (DFT) calculations reveal highly rearranged interfacial charges, yielding benefits for reducing the energy barriers of Na^+^ migration. The innovative NVMP‐NSRs with appropriate bulk defects exhibit considerable discharge capacity (120.1 mAh g^−1^ at 0.5C), high‐rate performance (70.9 mAh g^−1^ at 30C), and negligible capacity decay (3000 cycles at 20C). Moreover, the assembled NVMP‐NSRs//hard carbon full cells demonstrate a high energy density of 391.1 Wh kg^−1^ with excellent cyclic stability (91.2% after 100 cycles at 1C). The multiscale coupling strategy in this work offers new avenues to design high‐performance electrode materials toward fast electrochemical responses and robust structural stability.

## Graphical abstract

The poor electronic conductivity and intrinsic Jahn–Teller distortion in NASICON‐type Na_4_VMn(PO_4_)_3_(NVMP) led to insufficient capacity delivery and sluggish Na^+^ diffusion. Herein, a multiscale integration strategy is proposed to construct a unique 3D nanorod cathode enriched with V/O defects by utilizing DMF as a solvent. The crucial contribution of defects in modulating local unsaturated coordination states around vanadium atoms and the electronic structure of NVMP‐NRs. Meanwhile, enhanced contact between the cathode and electrolyte by nanorod morphology accelerates Na^+^ diffusion and excavates the electrochemical activity of the NASICON‐type cathode. This work provides an innovative avenue for the multiscale coupling of the defect and architecture engineering to promote ion/electrode transfer and shed light on designing high‐performance cathode materials in sodium‐ion batteries and beyond.

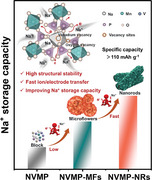

## 1 Introduction

Sodium‐ion batteries (SIBs) have been regarded as a highly cost‐effective option for grid‐scale energy storage systems due to the natural abundance and high availability of Na reserves.^[^ ^1^ ^]^ The synergistic targets of high energy density and a stable service lifespan of cathodes play a crucial role in commercial applications for SIBs.^[^ ^2^, ^3^ ^]^ In this regard, polyanionic materials, with unique inductive effects and multielectron reaction processes, have been deemed one of the most promising cathodes for SIBs, and their robust covalent framework endows them an edge over the competition.^[^ ^4^, ^5^ ^]^ Particularly, Na‐superior ion conductor (NASICON) structured Na_4_VMn(PO_4_)_3_ (NVMP) is attractive by virtue of its high specific capacity (110 mAh g^−1^) and suitable operating voltages (≈3.4 V for V^3+/4+^ and ≈3.6 V for Mn^2+/3+^ vs Na^+^/Na).^[^ ^6^ ^]^ Nevertheless, the poor electronic conductivity and intrinsic Jahn–Teller (J‐T) distortions of Mn^3+^ in NVMP lead to insufficient capacity delivery and sluggish Na^+^ diffusion, which severely restricts its further application.^[^ ^7^ ^]^ Tremendous efforts have been made to address these drawbacks, including carbonaceous materials coating,^[^ ^8^ ^]^ particle size reduction,^[^ ^9^ ^]^ and heterogeneous elements doping.^[^ ^10^ ^]^ Despite encouraging progress, the inevitable sacrifice of energy density, limited intrinsic conductivity, and complex synthesis methods warrant further improvement. Hence, in pursuing the synergistic ambitions of high energy density and stable lifespans, it is essential to explore visionary ways capable of delicately balancing these factors.

To date, defect engineering has drawn extensive attention as a promising strategy.^[^ ^11^, ^12^ ^]^ The distribution and local concentration of defects (e.g., vacancies, point defects, and dislocations) in the crystal lattice play a decisive role in modifying the electron delocalization structure, suggesting their superiority in accelerating charge transfer kinetics, and optimizing ion migration upon cycling.^[^ ^13^, ^14^, ^15^ ^]^ For instance, Hu et al.^[^ ^16^ ^]^ optimized the Li^+^ diffusion channels in *α*‐LiMn_0.5_Fe_0.5_PO_4_ nanocrystals by generating high concentrations of Fe^2+^‐Li^+^ antisite defects, achieving high‐rate capacity and good cycling stability. Qiao's group^[^ ^17^ ^]^ introduced vacancies into the transition metal layer of P2‐Na_0.7_Fe_0.1_Mn_0.75□0.15_O_2_ to suppress the migration of neighboring Na^+^, thereby maintaining structural and thermal stability in Na‐depleted states. Inspired by this, crystal structure tuning in NVMP by defect engineering modulates the surface electron transport properties, leading to the development of high‐rate performance, as well as mitigating volume changes induced by stress variations, thereby improving structural stability. However, few studies have been conducted on the role of defects in polyanionic materials, and their intrinsic mechanisms in electrochemical properties still remain ambiguous. Therefore, fully utilizing the potential of defect engineering to establish an explicit relationship between structure and properties could assist in improving the electronic conductivity and structural stability of intrinsic materials from a unique perspective.

In addition, the morphological design of NVMP particles is recognized as an active way to accelerate ion transport across the interface.^[^ ^18^ ^]^ Specifically, the implementation of nanomorphology could facilitate electrolyte penetration to increase the contact area with the electrode, thereby providing more activity for the redox reaction, while the short‐range diffusion path of nanoparticles confers Na^+^ a smaller diffusion resistance, thus improving Na^+^ diffusion kinetics.^[^ ^19^ ^]^ However, developing a new strategy to increase capacity, improve electronic conductivity, and enhance cycling stability remains a pressing challenge. Therefore, it is essential to sufficiently optimize the electrochemical properties of NVMP by multiscale coupling of bulk defects and architecture engineering.

**Figure 1:**
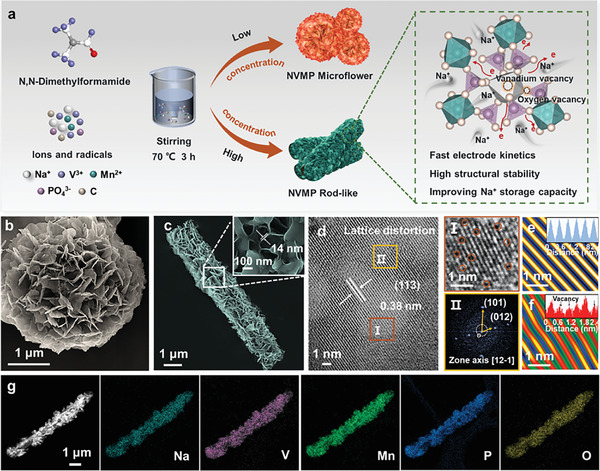
Synthesis and microstructure characterizations of NVMP‐NSFs and NVMP‐NSRs. a) Schematic diagram of the synthesis process, and SEM images for b) NVMP‐NSFs and c) NVMP‐NSRs. d) HRTEM images of NVMP‐NSRs. Corresponding FFT‐filtered atomic resolution images for e) NVMP‐NSFs and f) NVMP‐NSRs. g) Elemental mapping for NVMP‐NSRs.

Herein, we constructed a unique 3D NVMP nanosheet‐stacked rods cathode (denoted as NVMP‐NSRs) enriched with vanadium/oxygen (V/O) defects by regulating the nucleation process with a polar deprotonated solvent (DMF) (**Figure** **1**a). In this process, the surface energy of the NVMP crystal could be reduced by modulating the supersaturation degree in the solution to obtain morphologically controllable crystals, which allows highly effective contact with the electrolyte. X‐ray absorption spectroscopy (XAS) technologies reveal that V/O defects induce modifications in the coordination environment, including changes in the V‐O coordination number and bonding length, thereby altering the valence state and electronic structure of transition metal elements in NVMP‐NSRs. In addition, the local distortion caused by bulk defects in the crystal structure forcefully mitigates the negative deformation within MnO_6_ octahedra upon cycling, as well as utilizes space to relieve volume expansion. Integrated with theoretical calculations, the novel crystal defects act as electrochemically active sites to tune the local charge distribution at the interface and reduce the energy barriers of Na^+^ migration, synergistically enhancing Na^+^ transport kinetics. Thus, NVMP‐NSRs exhibit a high specific capacity (120.1 mAh g^−1^ at 0.5C), excellent rate performance (70.9 mAh g^−1^ at 30C), and long‐term durability (94.5% retention after 3000 cycles at 20C). This bulk‐defects and architecture engineering not only exceeds the trade‐off between fast kinetics and high stability of conventional cathode materials but also offers a new path for designing advanced energy storage materials.

## 2 Results and Discussion

The morphology evolution of the as‐prepared samples was characterized in detail by scanning electron microscopy (SEM). As shown in Figure S1 (Supporting Information), NVMP without the addition of DMF presents an irregular bulk morphology typical of polyanionic materials with a large size and uneven dispersion, which is detrimental to adequate contact between the cathode and electrolyte.^[^ ^20^ ^]^ While with DMF regulation, the growth pattern of NVMP altered, resulting in uniform multilayered hierarchical flower ball (NVMP‐NSFs) and rod‐like (NVMP‐NSRs) structures assembled from nanosheets, respectively (Figure 1b,c). Specifically, NVMP‐NSRs feature thinner and more uniform nanosheets with a size of ≈14 nm compared to NVMP (30 nm) (Figure S2, Supporting Information). It is ascribed to the electrophilic and nucleophilic nature of DMF, which acts as a source of intermediary components during the reaction dynamics and facilitates the dissolution‐recrystallization process.^[^ ^21^ ^]^ Furthermore, the surface area and pore size were further measured by the physical adsorption method (Figure S3, Supporting Information). The NVMP‐NSRs exhibit a large local pore size of ≈15 nm (Figure S3c, Supporting Information inset), on account of the unique 3D nanosheet‐stacked rod structure adapting to the stresses by generating voids and gaps.^[^ ^15^ ^]^ Based on the Brunauer–Emmett–Teller (BET) method, NVMP‐NSRs display the highest surface area of 27.4 m^2^ g^−1^ compared to NVMP‐NSFs (20.1 m^2^ g^−1^) and NVMP (11.2 m^2^ g^−1^). The multilayered porous structure effectively improves mass transfer and exposes more active sites than conventional polyanionic with irregular bulk morphology, and the increased surface area will facilitate homogeneous Na^+^ transport.^[^ ^21^ ^]^

To intuitively observe the microstructure of the as‐prepared samples, high‐resolution transmission electron microscopy (HRTEM) and corresponding energy‐dispersive spectroscopy (EDS) mapping were performed. From the HRTEM images, both NVMP‐NSFs and NVMP‐NSRs show a dense‐filled structure formed by layers of nanosheets stacked on top of each other, proving that the prepared materials are solid structures (Figure S4, Supporting Information). DMF is a highly polar solvent with strong coordination ability, which can interact with the precursors and influence the crystal growth process.^[^ ^22^ ^]^ As depicted in Figure 1d, the lattice fringes with a distinctive layer spacing of 0.38 nm are observed, corresponding to the (113) crystal plane of NVMP‐NSRs.^[^ ^23^ ^]^ Notably, the discontinuous lattice fringes within NVMP‐NSRs in the selected magnification region I (region marked by yellow circles) could be perceived as obviously internal structural defects. In addition, the selected area electron diffraction (SAED) was applied to analyze the bulk structure, since it was via a parallel electron beam to avoid high‐energy damage. As illustrated in the selected area marked as II, clear diffraction loops for (101) and (012) planes are observed,^[^ ^24^ ^]^ indicating that NVMP‐NSRs still maintain a well‐defined crystal structure. In contrast, as shown in Figure S5 (Supporting Information), NVMP shows clear lattice fringes with high crystallinity, indicating a near defect‐free feature. Figure 1e,f illustrates that NVMP‐NSRs display a significant lattice distortion compared to NVMP, where the structure is well maintained, and the complete lattice stripes are still clearly visible. The V/O defects in the NVMP‐NSRs structure provide more active sites for Na^+^ diffusion along the c‐axes, which exhibits immense potential for rapid energy storage applications.^[^ ^24^ ^]^ Subsequently, the corresponding energy spectrometer (EDS) elemental mappings unambiguously identify a uniform distribution of Na, V, Mn, P, and O elements in the as‐prepared samples (Figure 1g; Figure S6, Supporting Information). The carbon contents of NVMP, NVMP‐NSFs, and NVMP‐NSRs were characterized by thermogravimetric analysis (TGA), showing mass losses of 4.1, 4.4 and 4.5 wt.%, respectively (Figure S7, Supporting Information).

**Figure 2:**
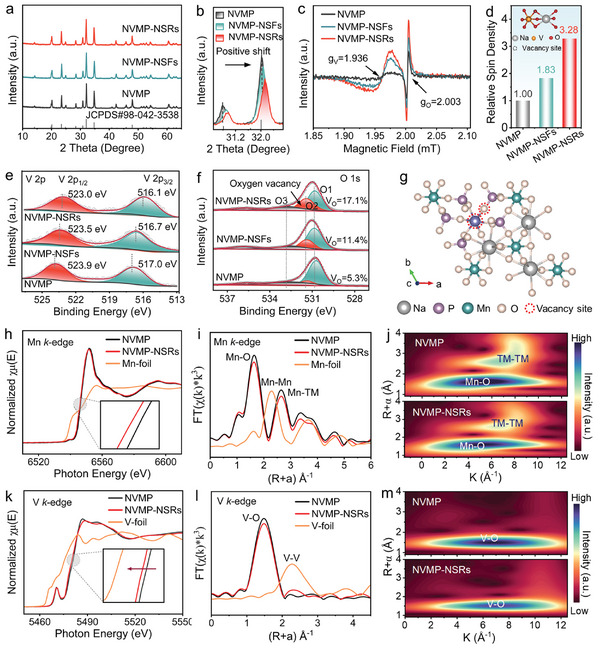
Electronic and local structural characterizations of the as‐prepared samples. a) XRD patterns and b) corresponding magnified image of (116) diffraction peak, c) EPR spectra and d) experimentally determined spin density, XPS spectra of e) V 2p and (f) O 1s for NVMP, NVMP‐NSFs and NVMP‐NSRs. g) Schematic diagram of the structure for NVMP‐NSRs along the *c*‐axes. h) Normalized Mn *K*‐edge EXAFS spectra, i) Mn *K*‐edge FT‐EXAFS in *R* space, and j) Wavelet transforms for the k^3^‐weighted EXAFS signals of NVMP and NVMP‐NSRs. k) Normalized V *K*‐edge EXAFS spectra, l) V *K*‐edge FT‐EXAFS in *R* space, and m) Wavelet transforms for the k^3^‐weighted EXAFS signals of NVMP and NVMP‐NSRs.

The crystal structures of the as‐prepared samples were analyzed using X‐ray diffraction (XRD) (**Figure** **2**a). All diffraction peaks could be well indexed with a rhombohedral *R* 3̅*c* space group (JCPDS No. 98‐042‐538),^[^ ^23^ ^]^ showing the samples possess high purity and good crystallinity. In contrast, the (112) and (116) facets of NVMP‐NSRs significantly broaden and shift to a higher angle, indicating that compressive strains are introduced following the formation of crystal defects (Figure 2b). As shown in Figure S8a (Supporting Information), the corresponding crystal structure of NVMP exhibits a 3D open framework consisting of corner‐shared TMO_6_ octahedra connected to PO_4_ tetrahedra through oxygen atoms. There are two kinds of independent Na sites in the NASICON structure: Na1 (6b) site and Na2 (18e) site, featuring six‐fold and eight‐fold coordination with oxygen atoms, respectively.^[^ ^25^ ^]^ More detailed cell parameters, such as accurate phase constituent as well as atomic arrangements are shown in Figure S8 (Supporting Information), and detailed cell parameters are summarized in Tables S1–S3 (Supporting Information). The results reveal that the lattice constants of NVMP‐NSRs slightly decrease with the introduction of crystal defects, which confirms the formation of some cation defects during the nucleation process of NVMP induced by DMF, resulting in lattice changes.

Electron paramagnetic resonance (EPR) spectroscopy was conducted to detect the vacancy species and concentration in NVMP‐NSRs (Figure 2c). Two pairs of symmetric peaks in the EPR signals centered at g = 1.936/2.003 could be assigned to unpaired electrons trapped around V/O defects in crystal lattice, separately.^[^ ^27^ ^]^ Notably, NVMP‐NSRs yield a stronger signal than NVMP and NVMP‐NSFs, implying a higher concentration of V/O vacancies. In addition, the relative spin density further demonstrates the highest defect content of NVMP‐NSRs among the three samples (Figure 2d), which may contain more active sites favoring Na^+^ storage. The detailed reasons reveal that the ligand bonds formed between the carbonyl group in the DMF molecule and the precursor metal ions significantly change the coordination environment of the metal ions and their reactivity, thus inducing lattice distortions or defects formation during the crystal growth stage.^[^ ^21^ ^]^ In addition, the gradual volatilization of DMF during the synthesis process was accompanied by the gradual removal of solvent molecules from the material, and this process triggered the creation of voids or stresses inside the material, which further contributed to the formation of defects. To further disclose the effect of V/O defects on structural stability, the chemical structure of the as‐prepared samples was analyzed by Raman spectroscopy. As shown in Figure S9a (Supporting Information), the peaks detected at 585.8 and 460.1 cm^−1^ correspond to the bending vibration modes of V─O bonds, while the low‐frequency peak at 337.8 cm^−1^ could be regarded as the bending vibration modes of V═O bonds.^[^ ^28^ ^]^ Specifically, with the increase of V/O defects, the Raman spectral peaks are shifted to lower wavenumbers (red‐shifted) due to the presence of the defects significantly affecting the vibrational modes and the chemical bond strength of the material. The combined effect of these factors led to a decrease in vibrational frequency, further confirming the destabilizing effect of V/O defects on the structural stability of the material, which is in accordance with the EPR results. In addition, the defects altered the chemistry of the surroundings, causing changes in the local electron cloud distribution. By comparing the Fourier transform infrared (FT‐IR) spectra of the three samples (Figure S9b, Supporting Information), it could be found that the vibration bonds of V─O (400≈600 cm^−1^) exhibit visible variation.^[^ ^26^, ^27^ ^]^ This change in the chemical environment is responsible for the change in the position of the peaks in the FT‐IR spectra by affecting the vibrational properties of the chemical bonds.

To examine the chemical composition and element valence states of the as‐prepared samples, X‐ray photoelectron spectroscopy (XPS) was performed. The XPS survey spectra in Figure S10a (Supporting Information) confirms the chemical compositions of NVMP, NVMP‐NSFs, and NVMP‐NSRs. As shown in Figure 2e, the high‐resolution V 2p spectra of NVMP are fitted into two broad peaks at 523.9 eV (V 2p_3/2_) and 517.0 eV (V 2p_1/2_), confirming the existence of V^3+^ species.^[^ ^8^ ^]^ Furthermore, the two peaks for NVMP‐NSRs (523.0 and 516.1 eV) shift to a lower binding energy region compared to NVMP and NVMP‐NSFs, implying higher electron‐cloud density and lower‐energy valence states in NVMP‐NSRs. The high‐resolution Mn 2p region shows the Mn 2p_3/2_ and Mn 2p_1/2_ spin‐orbit bands located at 654.5, 642.6, 653.2, and 641.3 eV, which could be attributed to Mn^3+^ and Mn^2+^, respectively (Figure S10b, Supporting Information).^[^ ^7^ ^]^ In contrast, NVMP‐NSRs feature a larger ratio of Mn^2+^/Mn^3+^ than NVMP, attesting to the different electronic structures caused by the defects. Additionally, in high‐resolution O 1s XPS spectra (Figure 2f), three sub‐peaks (O_1_, O_2,_ and O_3_) located at 530.8, 531.7, and 532.8 eV could be classified as lattice oxygen bonding with metal, deficient oxygen, and surface absorbed oxygen species, respectively.^[^ ^29^ ^]^ In detail, the characteristic peak of O_2_ in NVMP‐NSRs (17.1%) is higher than those in NVMP‐NSFs (11.4%) and NVMP (5.3%), demonstrating the successful introduction of defects with an arising oxygen coordination. To fully determine the difference between NVMP and NVMP‐NSRs with respect to oxygen defects, etched O 1s XPS was implemented along the 100 nm surface depth (Figure S11, Supporting Information). The signals of deficient oxygen for NVMP‐NSRs remain almost constant throughout the depth, while those for NVMP gradually decrease until they can't be detected in the interior. This suggests that oxygen defects are uniformly distributed in NVMP‐NSRs but are concentrated on the NVMP surface. Based on the above results, the atomic model for NVMP‐NSRs with V/O defects is illustrated in Figure 2g.

The detailed chemical state and local coordination environment of NVMP and NVMP‐NSRs were investigated by the X‐ray absorption fine structure (XAFS) measurements. As illustrated in the normalized Mn *K*‐edge X‐ray absorption near‐edge spectroscopies (XANES), the absorption edge of NVMP‐NSRs shifts toward lower energies compared to NVMP, implying a decreased average valence state in NVMP‐NSRs (Figure 2h). Consequently, there is a less positive charge state for Mn atoms and an electrically neutral system after the introduction of defects,^[^ ^30^ ^]^ which is consistent with the XPS results. Meanwhile, the Fourier transform extended X‐ray absorption fine structure (FT‐EXAFS) spectra were carried out to reveal the local structural rearrangement. From the Mn *K*‐edge in *R* space, NVMP‐NSRs show vividly decreased Mn‐O and Mn‐TM shell peaks than NVMP, demonstrating the reduced average O coordination numbers and less Mn ion aggregation around Mn (Figure 2i). Moreover, EXAFS fitting analysis was employed to quantitatively extract the structural parameters for Mn species, and the fitted curves matched well with the experimental spectra (Figure S12, Table S4, Supporting Information). The wavelet transforms (WT) of EXAFS spectra could be used to qualitatively identify and directly visualize the local bonding structure of metal atoms. As shown in Figure 2j, compared to NVMP, the maximum values of Mn‐O and Mn‐TM in WT images of NVMP‐NSRs appear at 5.1 and 6.0 Å^−1^, respectively, which are consistent with the results of FT‐EXAFS. As reflected in V *K*‐edge spectra (Figure 2k), the lower absorption edge energy indicates a lower average valence state in NVMP‐NSRs. Remarkably, the whine‐line peak energy of NVMP‐NSRs positively shifts ≈0.4 eV about NVMP, reflecting the effective electron transfer from the central V atom to neighboring atoms in NVMP‐NSRs inevitably triggers charge delocalization around the active site (Figure S13, Supporting Information).^[^ ^31^ ^]^ The weaker intensity of the white line peak further points to an enhanced electron density within the V 3d orbital due to the central atoms arrangement disorder in NVMP‐NSRs.^[^ ^32^ ^]^ In terms of the FT‐EXAFS spectra of V *K*‐edge in *R* space (Figure 2l), the characteristic peak at ≈1.51 Å is ascribed to the V‐O scattering in NVMP and NVMP‐NSRs, corresponding to the first shell of coordination.^[^ ^33^ ^]^ The intensity of the V‐O shell peak for NVMP‐NSRs conspicuously decreases in contrast to NVMP, elucidating the possible central V atom deficiency in NVMP‐NSRs. As well, EXAFS fitting results are provided in Figure S14 and Table S4 (Supporting Information), which are consistent well with the experimental spectra. It is noteworthy that the V atoms coordination numbers in NVMP‐NSRs are lower than that of NVMP, further verifying the existence of more unsaturated coordination structures in NVMP‐NSRs caused by bulk defects. Furthermore, the WT‐EXAFS images (Figure 2m) exhibit a decreased *K* position for the V─O bond, confirming the slightly altered chemical environment of V species in NVMP‐NSRs. Accordingly, the bulk defects within NVMP‐NSRs undoubtedly change the coordination states surrounding Mn and V atoms, further triggering charge delocalization and optimizing the *d*‐orbit electronic environment optimization on active sites, which is favorable for promoting charge transfer and multi‐electron conversion reaction kinetics, and thus endows NVMP‐NSRs with excellent sodium storage properties.

**Figure 3:**
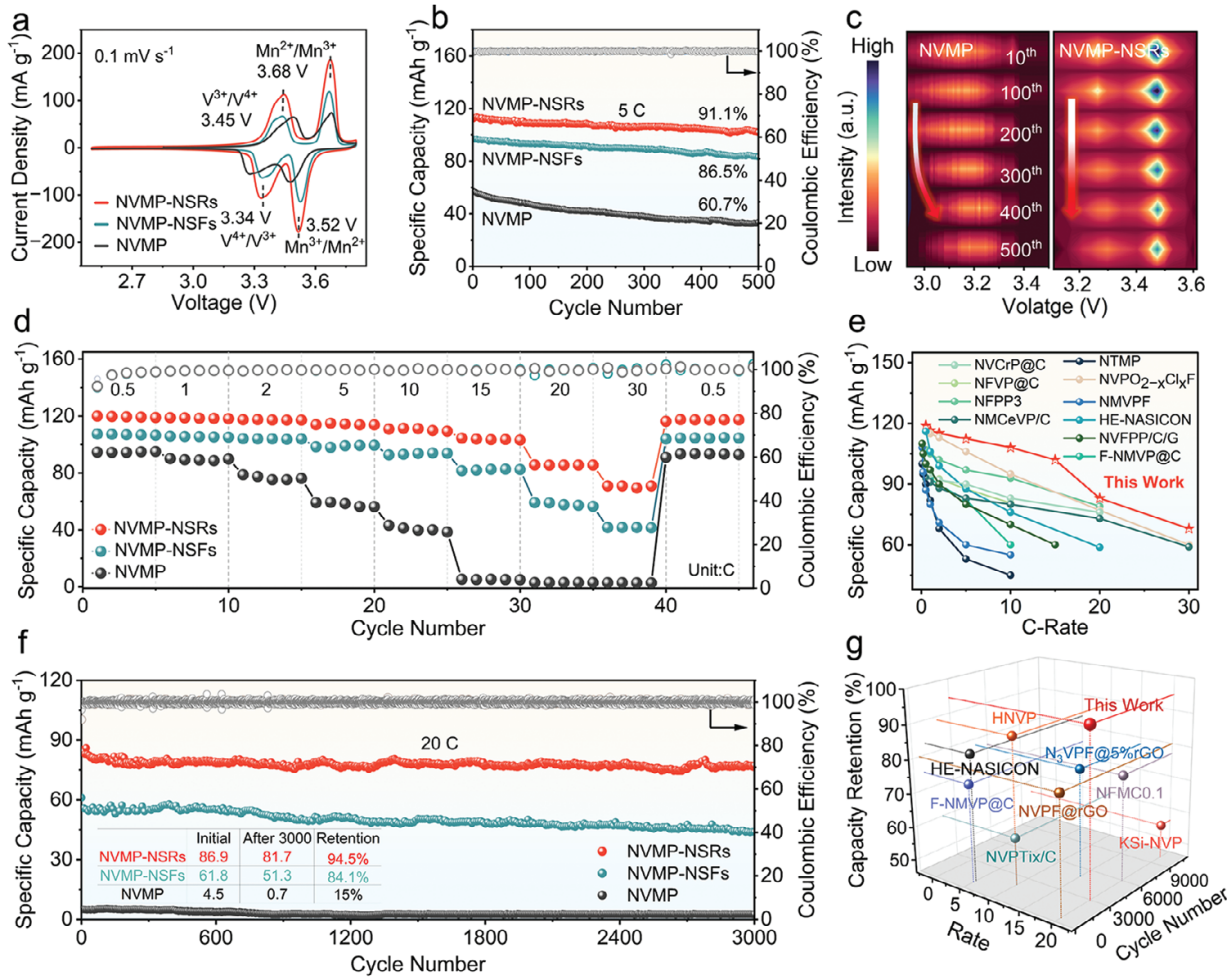
Electrochemical performances of NVMP, NVMP‐NSFs, and NVMP‐NSRs. a) CV curves at a scan rate of 0.1 mV s^−1^. b) Cycling performance at 5C and c) the corresponding dQ/dV curves from the 10th to 500th cycles of NVMP and NVMP‐NSRs. d) Rate capabilities at the C‐rate ranging from 0.5C to 30C. f) Long‐term cycling performance at 20C. e) Comparison of rate properties and g) capacity retention in this work to previously reported phosphate cathodes.

In view of the obvious electronic/local structural modifications resulting from bulk defects and architecture engineering in NVMP and NVMP‐NSRs, excellent charge carrier storage abilities and Na^+^ transportation are brought about, which may further enhance the performance of SIBs. Therefore, the electrochemical performances of the as‐prepared samples were evaluated in Na‐ion half‐cells by galvanostatic charge/discharge (GCD) and cyclic voltammetry (CV) tests, covering the voltage window of 2.5–3.8 V (vs. Na^+^/Na). As presented in CV curves (**Figure** **3**a), two pairs of redox peaks are located at 3.34/3.45 and 3.52/3.68 V, which corresponds to the redox reactions of V^3+^/V^4+^ and Mn^2+^/Mn^3+^ coupling,^[^ ^21^ ^]^ respectively. Notably, the smallest voltage gap between the oxidation and reduction peaks of NVMP‐NSRs signifies the lowest polarization and the highest current intensity compared to NVMP and NVMP‐NSFs indicate the most outstanding electrochemical activity, guaranteeing better reversibility upon Na^+^ de‐/insertion. As plotted in Figure S15 (Supporting Information), the galvanostatic charge/discharge profiles for the first cycle of all samples were collected at 0.5C (1C = 110 mAh g^−1^). The NVMP‐NSRs deliver a discharge capacity of 120.1 mAh g^−1^ with ≈100% coulombic efficiency, presenting a two‐step Na^+^ de‐/insertion process, which coincident well with CV curves. By contrast, the initial discharge capacities of NVMP‐NSFs and NVMP are 107.4 and 94.6 mAh g^−1^ at 0.5C, respectively. The substantially increased capacity of NVMP‐NSRs could be attributed to the visibly short diffusion pathways and sufficient contact between the electrode and electrolyte under the unique 3D nanosheet‐stacked rods architecture, coupled with the enhanced pseudocapacitive behavior (easier Na^+^ surface adsorption with lower binding energies) enabled by the formation of more defect sites, thus providing a better Na^+^ storage performance (to be introduced and discussed later). Especially, the combination of considerable capacity with a high average output voltage (≈3.45 V) endows the electrode with an impressive energy density of 413.1 Wh kg^−1^.

The cycling stability of the three electrodes was investigated in Figure 3b at 5C. The reversible capacity of NVMP‐NSRs remains at 102.5 mA h g^−1^ after 500 cycles with a higher capacity retention of 91.1% than that of NVMP‐NSFs (84.4 mAh g^−1^, 86.5%) and NVMP (33.3 mAh g^−1^, 60.7%), suggesting the superior structural stability and highly reversible Na^+^ de‐/insertion kinetics. Furthermore, the corresponding dQ/dV curves of NVMP‐NSRs render much well‐maintained redox peaks compared to NVMP even after 500 cycles (Figure 3c), which could be attributed to the V/O defects and morphological regulation to promote the charge transfer and enhance structural stabilization. The enhanced Na^+^ storage capacity for NVMP, NVMP‐NSFs, and NVMP‐NSRs is further proved by the rate capabilities in Figure 3d. In which NVMP‐NSRs manifest excellent rate performance with discharge capacities of 120.1, 118.9, 117.5, 115.1, 111.4, 103.9, 85.5, and 70.9 mA h g^−1^ at 0.5, 1, 2, 5, 10, 15, 20, and 30C, respectively. The excellent high‐rate performance of NVMP‐NSRs arises from the increased electron concentration due to the defect sites serving as shallow donors to guarantee rapid Na^+^ de‐/insertion during cycling at ultrahigh rates, rendering superiority over other reported Mn‐based SIB cathodes (Figure 3e).^[^ ^7^, ^8^, ^10^, ^34^, ^35^, ^36^, ^37^, ^38^, ^39^, ^40^, ^41^ ^]^ After 70 cycles, the reversible specific capacity rapidly restores to its initial value as the *C*‐rate returns to 0.5C, attesting to the exceptional rates capability for Na^+^ storage in NVMP‐NSRs, which is higher than that of NVMP and NVMP‐NSFs. Specifically, the discharge capacity of NVMP rapidly fades to only 5.5 mAh g^−1^ when the current density increases to 30C. As shown in Figure S16 (Supporting Information), the shape retention of GCD profiles for NVMP‐NSRs at different *C*‐rates also maintains a better condition compared to other samples. Subsequently, the long‐term cycling durability at a high *C*‐rate of 20C for NVMP‐NSRs was conducted for further evaluation in Figure 3f. NVMP‐NSRs deliver a high discharge capacity of 86.9 mAh g^−1^ and maintain at 81.7 mAh g^−1^ after 3000 cycles, with a capacity retention of 94.5%, which is distinctly better than those of NVMP‐NSFs (51.3 mAh g^−1^, 84.1%) and NVMP (0.7 mAh g^−1^, 15%). Overall, compared to NVMP, the superior electrochemical performances in NVMP‐NSRs suggest powerful evidence for structural robustness in favor of the Na^+^ de‐/insertion process during cycling. More importantly, the significant performance in capacity retention of NVMP‐NSRs is preferable to that of most previously reported phosphates cathode materials in SIBs (Figure 3g).^[^ ^8^, ^36^, ^37^, ^42^, ^43^, ^44^, ^45^, ^46^, ^47^ ^]^

**Figure 4:**
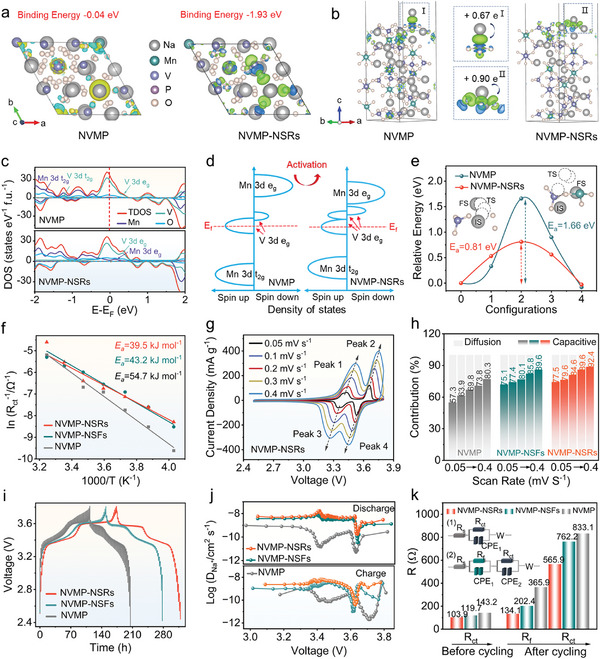
Electronic structure evolution and reaction kinetics analyses of the as‐prepared samples. a) The adsorption energy for Na^+^ and b) charge density difference distribution in NVMP and NVMP‐NSRs. c) The total density of states, and d) the corresponding schematic DOS of NVMP and NVMP‐NSRs. e) Calculated Na^+^ diffusion energy barriers for NVMP and NVMP‐NSRs. The insets in e show the Na^+^ diffusion pathways. f) The diffusion activation energy compared with NVMP, NVMP‐NSFs, and NVMP‐NSRs. g) CV curves at various scan rates of NVMP‐NSRs. h) Pseudocapacitive contributions at various scan rates, i) GITT curves, and j) the corresponding Na^+^ diffusion coefficient of the as‐prepared samples. k) Comparison of *R*_ct_ values at different states of the as‐prepared samples.

To investigate the impact of V/O defects on the electrochemical kinetics as well as the intrinsic properties of their rhombic structure stability in NVMP‐NSRs, density functional theory (DFT) calculations were implemented. First, the most stable atomic arrangements of the 1 × 1 × 1 supercell without and with different defect geometrical configurations were simulated and presented in Figure S17 (Supporting Information). In addition, based on the adsorption energy (Δ*E*_a_) results in **Figure** **4**a, NVMP‐NSRs display a stronger Na^+^ adsorption in the vicinity of defect sites with a lower Δ*E*_a_ (−1.93 eV) than that of NVMP (−0.04 eV), implying the defect sites provide effective adsorption for the increased surface‐induced pseudocapacitive charge storage. Accordingly, the charge distribution in both NVMP and NVMP‐NSRs was investigated by surface electron density difference modeling (Figure 4b). Remarkably, the coexistence of V/O defects in NVMP‐NSRs induces distinct interfacial charge redistribution compared to NVMP, indicative of strengthened interactions between Na^+^ and surface materials, leading to higher redox reactivity, in agreement with the enhanced binding‐driven Na^+^ adsorption. Subsequently, the density of states (DOS) and diffusion barrier for Na^+^ migration of NVMP and NVMP‐NSRs was further simulated to evaluate the effectiveness of the V/O defects. As displayed in Figure 4c, the total density of states (TDOS) of transition metal 3*d* orbitals and oxygen 2*p* orbitals in NVMP and NVMP‐NSRs, and the schematic band diagrams were plotted in Figure 4d. It is noteworthy that both NVMP and NVMP‐NSRs manifest metallic properties, where the V *e*_g_ orbital of NVMP‐NR splits into two peaks, showing a higher TDOS value than that of NVMP, which is a favorable validation for effectively improving the intrinsic electron conductivity.

Additionally, the Na^+^ migration path in NVMP and NVMP‐NSRs was also explored (Figure 4e), where IS represents the initial state, TS represents the transition state, and FS represents the final state (inset in Figure 4e). To be specific, the values of the energy barrier dropped from 1.66 eV (NVMP) to 0.81 eV (NVMP‐NSRs), indicating more favorable migration behavior of Na^+^ in NVMP‐NSRs. This significantly lower diffusion barrier of NVMP‐NSRs suggests the de‐/insertion of Na^+^ in NVMP‐NSRs is much more easily affected by the bulk defects, which could significantly improve the electrochemical properties of NVMP‐NSRs. As well, the much lower diffusion energy barrier in NVMP‐NSRs reveals a more inclined Na^+^ escape capability thus providing another compelling evidence to explain the excellent reaction kinetics and desirable rate performance in NVMP‐NSRs.

Further probe into Na^+^ diffusion kinetics was conducted to compare the influence on Na‐storage properties of electrodes. The Nyquist plots of the as‐prepared samples were recorded at varying temperatures (−25≈35 °C) (Figure S18, Supporting Information), in which the activation energy (*E*_a_) of NVMP‐NSRs (39.5 kJ mol^−1^) is found to be lower than that of NVMP (43.2 kJ mol^−1^) and NVMP‐NSFs (54.7 kJ mol^−1^), indicating a lower desolvation activation energy and a larger proportion of activated molecules. This substantiates that defect engineering could facilitate the separation of the solvated Na^+^ sheath layer and promote the fast intercalation kinetics of Na^+^ (Figure 4f). In addition, the CV measurements were systematically employed at different sweep rates ranging from 0.05 to 0.4 mV s^−1^ to distinguish Na^+^ storage kinetics, as shown in Figure 4g and Figure S19a (Supporting Information). Visually, the CV curves of NVMP‐NSRs feature a similar shape with increasing sweep rates, compared to NVMP, demonstrating a small polarization voltage. The *b* values for the four redox peaks of NVMP and NVMP‐NSRs are determined to be between 0.5 and 1.0, suggesting that the Na^+^ storage process is controlled by a synergistic interaction between ion diffusion and capacitance characteristics (Figure S19, Supporting Information). As shown in Figure 4h, the capacitive contribution of NVMP‐NSRs at the scan rate of 0.05, 0.1, 0.2, 0.3, and 0.4 mV s^−1^ were calculated to 77.5%, 79.6%, 84.6%, 89.6%, and 92.4%, respectively, significantly higher than those of NVMP and NVMP‐NSFs. The gradually increased capacitive contribution percentage demonstrates that this directly affects rate performance because of the fast Na^+^ kinetics. The galvanostatic intermittent titration technique (GITT) was carried out to disclose a dynamic changing tendency for coefficients *D*_Na_^+^ during Na^+^ de‐/insertion throughout the entire cycling process (Figure 4i). Partial test diagrams during charge and discharge progress are provided in Figure S20 (Supporting Information). Notably, the two modified samples showed no sign of kinetic performance degradation near the voltage of 3.4 V, and their excellent kinetic stability is tightly related to the unique structural properties of the materials. It is attributed to the special structure formed by the unique nanosheet stacking which enhances the wettability of the electrolyte and exposes more active sites, simultaneously optimizes the Na^+^ transport path and reduces the interfacial impedance, thus maintaining the efficient Na^+^ diffusion kinetics.^[^ ^3^, ^48^, ^49^ ^]^ The results show that NVMP‐NSRs exhibit the highest *D*_Na_^+^ values among the three samples, specifically, the *D*_Na_^+^ of NVMP‐NSRs is ≈10 times greater than that of NVMP (Figure 4j), uncovering the rapid Na^+^ diffusion property in NVMP‐NSRs. In addition, the charge‐transfer resistance was also revealed by electrochemical impedance spectroscopy (EIS) analysis (Figure S21, Supporting Information). Based on the equivalent circuit provided in Figure 4k, the EIS curves are well‐fitted. It is found that the values of charge transfer resistance (*R*_ct_) for NVMP‐NR are the smallest before and after cycling, which indicates the augmented electrode kinetics and accelerated ion conversion kinetics, proving that the superior rate performance in NVMP‐NR could be attributed to the advanced performance of the defect and architecture engineering. Following the identification of the lower spatial diffusion resistance, NVMP‐NSRs illustrate excellent high‐loading chemical performances. As shown in Figure S22a (Supporting Information), NVMP‐NSRs show an excellent capacity retention of 92.2% for 100 cycles at 0.5C under a loading of 2.1 mg cm^−^^2^. Also, at a loading of 7.1 mg cm^−^^2^, the capacity retention of NVMP‐NSRs is still able to maintain a high level of 88.3% after 100 cycles at 0.5C, indicating an efficient Na^+^ de‐/insertion (Figure S22b,c, Supporting Information). Relevant detailed electrochemical performance tests are further discussed in the supporting information (in Figure S22, Supporting Information), indicating the as‐proposed bulk‐defects and architecture engineering could not only optimize crystal structure for phosphate with spatial and conductivity merits but also enhance the potential for practical application in SIBs.

**Figure 5:**
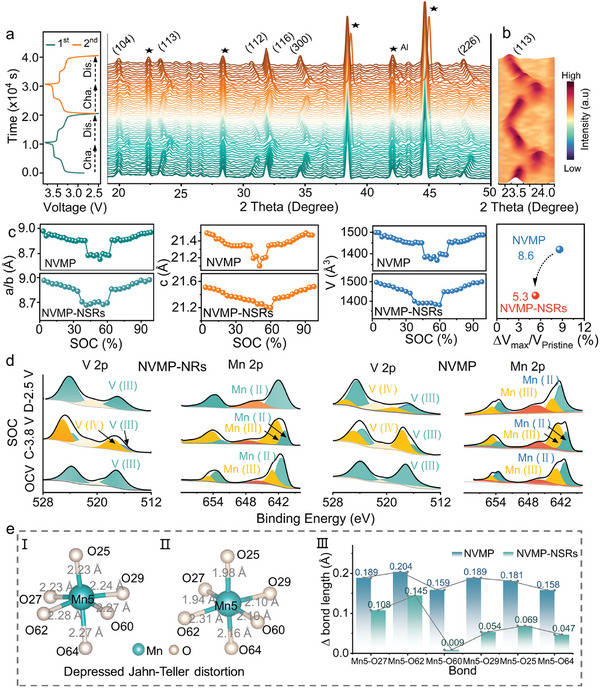
Structure evolution and charge compensation of the as‐prepared electrodes. a) In situ XRD patterns of NVMP‐NSRs collected at the selected 2*θ* range. b) The 3D contour maps of (113) XRD peak evolution in NVMP‐NSRs. c) Unit cell parameters evolution during Na^+^ de‐/insertion for NVMP‐NSRs. d) Ex situ XPS of high‐resolution Mn 2p and V 2p spectra. e) The schematic illustrations of structural geometric in MnO_6_ octahedral for NVMP (I) and NVMP‐NSRs (II), and (III) the variation of Mn─O bond length at the discharged state of NVMP and NVMP‐NSRs caused by J‐T distortion.

The detailed structural evolution of two cathodes during Na^+^ de‐/insertion was monitored through in situ XRD. **Figures** **5**a and S23 (Supporting Information) present the 2D plot of the in situ XRD patterns for NVMP‐NSRs and NVMP during the first two cycles, with the corresponding GCD curves obtained between 2.5 and 3.8 V at 0.2C. Notably, in the initial stage, the (104), (113), (112), (116), (300), and (226) characteristic peaks are significantly shifted to a higher degree and gradually weaken upon charging to 3.5 V, indicating a typical solid‐solution reaction mechanism based on V^3+^/V^4+^ redox.^[^ ^10^ ^]^ Afterward, the appearance of new characteristic diffraction from 3.5 to 3.8 V implies the formation of a new phase (Figure 5b), demonstrating the biphasic reaction mechanism based on Mn^2+^/Mn^3+^ redox.^[^ ^50^ ^]^ Meanwhile, the crystal cell parameters *a*/*b* and *c* axes decrease with Na^+^ extraction, corresponding to the shrinkage in lattice volume. All characteristic peaks recover to low angles, and the *a*/*b* and *c* axes return to the initial values during discharge, validating reversible cell respiration. Based on the Rietveld refinement results, the volume shrinkage/expansion of NVMP‐NSRs was calculated to be 5.3% (△*V*_max_/△*V*_pristine_), much smaller than that of NVMP (8.6%) upon the first Na^+^ de‐/insertion, unambiguously demonstrating the ideal structural stability of NVMP‐NSRs (Figure 5c). Moreover, the structural evolution of NVMP‐NSRs upon the charging/discharging process was further confirmed by HRTEM images. As depicted in Figure S24 (Supporting Information), the (113) lattice plane space shrinks from 0.38 to 0.37 nm, and then completely recovers to the initial state, illustrating the highly reversible shrinkage/expansion of interplanar spacing during the Na^+^ de‐/insertion in the first cycle. The results of in situ XRD and HRTEM primarily support the idea that the V/O defects could stabilize the framework and inhibit structural distortion of NVMP‐NSRs, which is a key factor in ensuring long‐term cycling stability.

The stepwise redox reaction of NVMP and NVMP‐NSRs was systematically unveiled by ex situ XPS analyses (Figure 5d). In the initial state, the peaks located at 522.9 eV and 516.1 eV in V 2p belong the V 2p_1/2_ and V 2p_3/2_, respectively, affirming the trivalent state of vanadium.^[^ ^51^ ^]^ While the Mn 2p_1/2_ and Mn 2p_3/2_ peaks located at 641.5, 653.3, and 643.3, 654.8 eV imply the coexistence of divalent manganese and trivalent manganese in the pristine material.^[^ ^10^ ^]^ Upon charging to 3.8 V, the peaks of V 2p shift toward higher binding energy, which corresponds to the oxidation process of V^3+^ to V^4+^ during the Na^+^ insertion into NVMP‐NSRs. At the same time, the Mn 2p is conspicuously moved to higher binding energies, validating a completely oxidized Mn^3+^. With a subsequent discharge from 3.8 to 2.5 V, the V 2p and Mn 2p XPS spectra of NVMP‐NSRs both fully return to their original states, while the visualized irreversible transformation of V and Mn emerges in NVMP. These results demonstrate the excellent Na^+^ storage reversibility of NVMP‐NSRs.

To shed further light on the structural stability of NVMP and NVMP‐NSRs, the influence of J‐T distortion of Mn(III)‐O_6_ octahedron and structural changes was examined. Generally, the geometric structure distortion is mainly contributed to the shrinkage or elongation of the O─Mn─O bond along the axes direction in the octahedral units.^[^ ^8^ ^]^ Figure 5e I,II provide the MnO_6_ octahedron models of NVMP and NVMP‐NSRs, respectively, with a detailed comparison of the Mn─O bond length variation upon Na^+^ de‐/insertion. After the first cycle, the crystal distortion of NVMP‐NSRs is significantly suppressed with a faint change in the length of Mn─O bonds (0.009 Å for Mn5‐O60) (Figure 5e III). By contrast, due to the severe J‐T distortion suffered by Mn^3+^ in NVMP, serious stretching and compression of the Mn─O bond occurs. Overall, the NVMP‐NSRs framework is well maintained after bulk defects as well as architecture engineering‐induced special structures.

**Figure 6:**
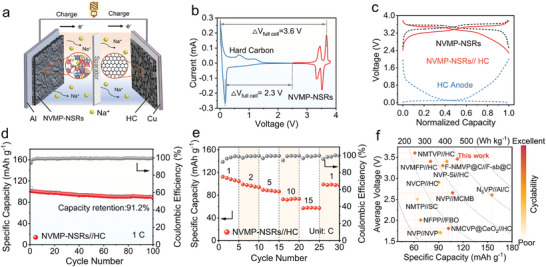
Electrochemical performances of NVMP‐NSRs//HC full cell. a) Schematic illustration of the full cell. b) CV curves of NVMP‐NSRs cathode and HC anode. c) GCD profiles of NVMP‐NSRs cathode, HC anode, and the full cell. d) Cycling performance and e) rate capabilities of the full cell. f) A comparison of the full cell with other reported SIBs in terms of specific capacity and average voltage.

To further elucidate the practicability of NVMP‐NSRs, the sodium‐ion full cells were constructed by pairing the NVMP‐NSRs cathode with the commercial hard carbon (HC) anode (**Figure** **6**a). The electrochemical performance of the HC anode was predicted in a half cell before the formal matching, which could maintain a stable reversible capacity of ∼317.5 mAh g^−1^ under the current density of 100 mA g^−1^ during cycling (Figure S25, Supporting Information). Based on the CV curves of NVMP‐NSRs cathode and HC anode (Figure 6b), the voltage window for full cell was determined to be 2.3–3.6 V and the active mass ratio of cathode to anode was controlled to 3:1. The GCD profiles of voltage versus normalized capacity for NVMP‐NSRs cathode, HC anode, and the full cell were illustrated in Figure 6c, where the shape and voltage of the GCD profiles for full cell were well maintained, demonstrating the perfect matching between cathode and anode. Moreover, an initial discharge capacity of 111.7 mAh g^−1^ at 1C along with an average operating voltage of ≈3.5 V indicates the NVMP‐NSRs//HC full cell could achieve a maximum specific energy density of 391.1 Wh kg^−1^ (based on the NVMP‐NSRs mass). As depicted in Figure 6d, the full cells could retain high reversible capacities of 90.9 mAh g^−1^ at 1C after 100 cycles, with promising capacity retentions of 91.2%. Moreover, the full cells also exhibit an excellent rate performance with high discharge capacities of 110.4, 97.9, 88.6, 73.2, and 58.1 mAh g^−1^ at 1, 2, 5, 10, and 15C, respectively (Figure 6e). When compared to the representative Na^+^ full cells reported in the literature (Figure 6f), the NVMP‐NSRs//HC full cell manifests competitive average discharge voltage, rate capability, and cyclability.^[^ ^8^, ^37^, ^42^, ^43^, ^52^, ^53^, ^54^, ^55^, ^56^, ^57^ ^]^ The comprehensive electrochemical performances further confirm the application potential of NVMP‐NSRs for future high‐performance energy storage devices.

## 3 Conclusion

In summary, bulk‐defect‐rich, 3D nanosheet‐stacked rods NVMP‐NSRs with high‐rate capability and cycling stability were proposed for SIBs. The local coordination environment and electronic structure of NVMP‐NSRs are effectively regulated via V/O defects, which significantly suppress the lattice distortion upon cycling. Moreover, the nanosheet‐stacked rod morphology increases contact between the cathode and electrolyte, accelerates Na^+^ diffusion, and strengthens pseudocapacitive behaviors. Specifically, XAS characterizations certify the defect‐induced variations in V─O bonding distance and coordination number for NVMP‐NSRs, which is beneficial for exposing more unsaturated coordinative sites. DFT calculations reveal that defective structures enriched within NVMP‐NSRs could function as readily accessible electrochemical activity origins, participating in regulating the inherent charge rearrangement and reducing the Na^+^ migration barrier, thereby improving ion transport kinetics. Furthermore, in situ, XRD, and in‐depth studies of ion migration kinetics show that NVMP‐NSRs endorse both slight volume change and fast ion migration rate during Na^+^ de‐/insertion. Consequently, the NVMP‐NSRs deliver a high specific capacity (120.1 mAh g^−1^ at 0.5C), excellent rate performance (70.9 mAh g^−1^ at 30C), an ultrastable cycling property (94.5% capacity retention after 3000 cycles at 20C), and satisfactory full cell performances when assembled with HC anode. This work provides an innovative avenue for the multiscale coupling of bulk defects and architecture engineering to accelerate Na^+^ diffusion kinetics for high‐performance electrode materials.

## Conflict of Interest

The authors declare no conflict of interest.

## Supporting information



Supporting Information

## Data Availability

The data that support the findings of this study are available from the corresponding author upon reasonable request.
