# Supplementary material for: Accelerating Electrochemical Responses of Na4VMn(PO4)3 via Bulk‐Defects and Architecture Engineering for High‐Performance Sodium‐Ion Batteries
Source: Adv Sci (Weinh). 2025 Apr 17;12(21):2415331. doi: 10.1002/advs.202415331 (PMC12140301; doi:10.1002/advs.202415331)
Supplement: Supplementary file 1 — Supporting Information [file ADVS-12-2415331-s001.docx]

**Supporting Information**

**Accelerating Electrochemical Responses of Na_4_VMn(PO_4_)_3_ via Bulk-defects and Architecture Engineering for High-performance Sodium-ion Batteries**

*Jingwen Zhao^a#^, Bo Zou^c#^, Weitao Yan^b^, Shijia Li^a^,* *Wentao Wu^b^, Wei-Hua Wang^b^, Shiyu Li^a,^*, and Ying Bai^a,^**

^a^Key Laboratory for High Efficiency Energy Conversion Science and Technology of Henan Province, International Joint Research Laboratory of New Energy Materials and Devices of Henan Province, School of Physics and Electronics, Henan University, Kaifeng, 475004, P. R. China

^b^Department of Electronic Science and Engineering, Tianjin Key Laboratory of Photo-Electronic Thin Film Device and Technology, Engineering Research Center of Thin Film Optoelectronics Technology (Ministry of Education), Nankai University, Tianjin 300350, China

^c^School of Science and Ministry of Industry and Information Technology Key Laboratory of Micro-Nano Optoelectronic Information System, Harbin Institute of Technology, Shenzhen, 518055, China

*Corresponding author. E-mail: Li-SY@henu.edu.cn (S. Y. Li); ybai@henu.edu.cn (Y. Bai)

**Experimental Procedures**

***Synthesis*:** All the regents with analytical purity were purchased from Shanghai Aladdin Biochemical Technology Co., Ltd. Na_4_VMn(PO_4_)_3_ (NVMP) were synthesized via a sol-gel method followed by an annealing treatment under Ar atmosphere. Typically, the raw materials including sodium carbonate (Na_2_CO_3_), vanadium pentoxide (V_2_O_5_), manganese acetate tetrahydrate (Mn(CH_3_COO)_2_·4H_2_O), and ammonium biphosphate (NH_4_H_2_PO_4_) with a stoichiometric amount of 4:1:1:3, together with a certain amount of anhydrous citric acid (C_6_H_8_O_7_) as a chelating agent and carbon source were dissolved in 70 mL of deionized (DI) water by a water-bath heating process. The NVMP-NSFs and NVMP-NSFs precursors were obtained in the same way as NVMP by adding different masses of DMF. After further stirring for 6 h, the gel was dried at 60°C in a vacuum oven for 24 h. Finally, the obtained precursors were annealed at 750°C for 9 h in an Ar atmosphere to obtain a series of cathode materials. We immediately transferred these samples to a glovebox to avoid exposure to air after sintering.

***Characterizations*:** The surface morphology and energy-dispersive spectroscope (EDS) were observed by the scanning electron microscope (SEM, JEOLJSM-7610FPlus). The fine microstructure of samples was detected by a high-resolution transmission electron microscope (HR-TEM, JEOLJEM-F200). The crystal structure of samples was characterized by X-ray diffractometer (XRD, Cu Kα, Smartlab-9). The XRD refinement was performed by the VESTA software. The X-ray photoelectron spectroscopy (XPS, Al, KαESCALAB250Xi) was employed to determine the elements valence and structural information of samples and electrodes. Carbon content of samples was analyzed by thermogravimetric analyzer (TGA, DTA6300). Brunauer-Emmett-Teller (BET) specific surface area and pore size distribution of samples were measured by nitrogen adsorption-desorption curves. The detailed structure features of samples were further characterized by Raman spectra (Laser GS6000) and Fourier-transform infrared spectroscopy (FTIR, T27). The *in situ* XRD experiment was performed on an X-ray diffractometer equipped with a Cu Kα radiation (λ = 1.5406 Å).

***Electrochemical measurements*:** For fabricating the working electrodes, active material, Super-P, and polyvinylidene fluoride (PVDF) binder were mixed in a ratio of 7:2:1, with N-methyl-2-pyridinone (NMP) as the solvent to form a slurry to prepare the desired electrode. Hard carbon electrode consisted of commercial hard carbon, acetylene black, and polyvinylidene fluoride (PVDF) in a mass ratio of 9:0.5:0.5. In half-cells, the 2032 coin-type cells were assembled in the argon-filled glovebox, employing Na metal as counter, glass fiber as separator and 1 M NaClO_4_ in propylene carbonate (PC) with 2% fluoroethylene carbonate (FEC) additive as electrolyte. For full-cell assembly, the previously prepared active material electrode was used for the positive electrode, while a hard carbon electrode was used for the negative electrode. The electrolyte was the same as that used in the half-cell, i.e., 1 M NaClO_4_ dissolved in PC solvent containing 2% FEC additive. Prior to full battery assembly, the HC anode was pre-cycled in a half cell at 50 mA g^-1^ within a voltage window from 0.01 to 2.0 V (vs. Na^+^/Na). Subsequently, the anode was disassembled and matched with the NVMP-NSRs cathode. The full cells were measured within the voltage windows of 2.3-3.6 V, and the corresponding capacity ratios of cathode to anode was about 1.1~1.3. The amount of electrolyte was 100 μL. The Na^+^ storage performance was evaluated on the LAND battery test system (CT2001A). Cyclic voltammetry (CV) and electrochemical impedance spectroscopy (EIS) tests with a frequency range of 100 kHz to 0.01 Hz were performed on an electrochemical workstation (CHI660E). The static galvanostatic intermittent titration technique (GITT) used a unique GCD mode, and its duty cycle consisted of two phases: the charge/discharge process lasted 40 minutes at 0.1 C, followed by a 2 h pause. The voltage window for the cell was within 2.5-3.8 V, specially, the nominal capacity of 1 C was designated as 110 mA g^-1^.

***Calculation Details*:** The geometrical and electronic structures of pristine Na_4_VMn(PO_4_)_3_ and defect systems have been investigated by adopting Vienna Ab-initio Simulation Package (VASP) code based on density functional theory (DFT). The generalized gradient approximation with Perdew-Burke-Ernzerhof (GGA-PBE) format was used to describe the exchange correlation potential.^[1]^ The (001) surface of Na_4_VMn(PO_4_)_3_ was adopted to study the adsorption of Na^+^. The optimization of atomic positions stopped until the force on each atom was less than 0.05 eV Å^-1^. The energy convergence criterion was 10^-4^ eV. The cutoff energy was 400 eV in all the calculations. The *k*-point grid of 1×1×1 was adopted for pristine Na_4_VMn(PO_4_)_3_ and defect systems, respectively. The energy barriers of Na^+^ ions diffusion in the (001) surface of pristine Na_4_VMn(PO_4_)_3_ and defect systems were calculated through the climbing image nudged elastic band (CI-NEB) method.^[2]^

**Results and Discussion**

**Figure S1.** SEM images of (a, d) NVMP, (b, e) NVMP-NSFs and (c, f) NVMP-NSRs.


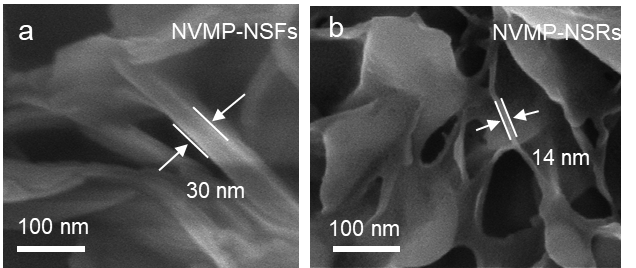


**Figure S2.** Enlarged SEM images of (a) NVMP-NSFs and (b) NVMP-NSRs.


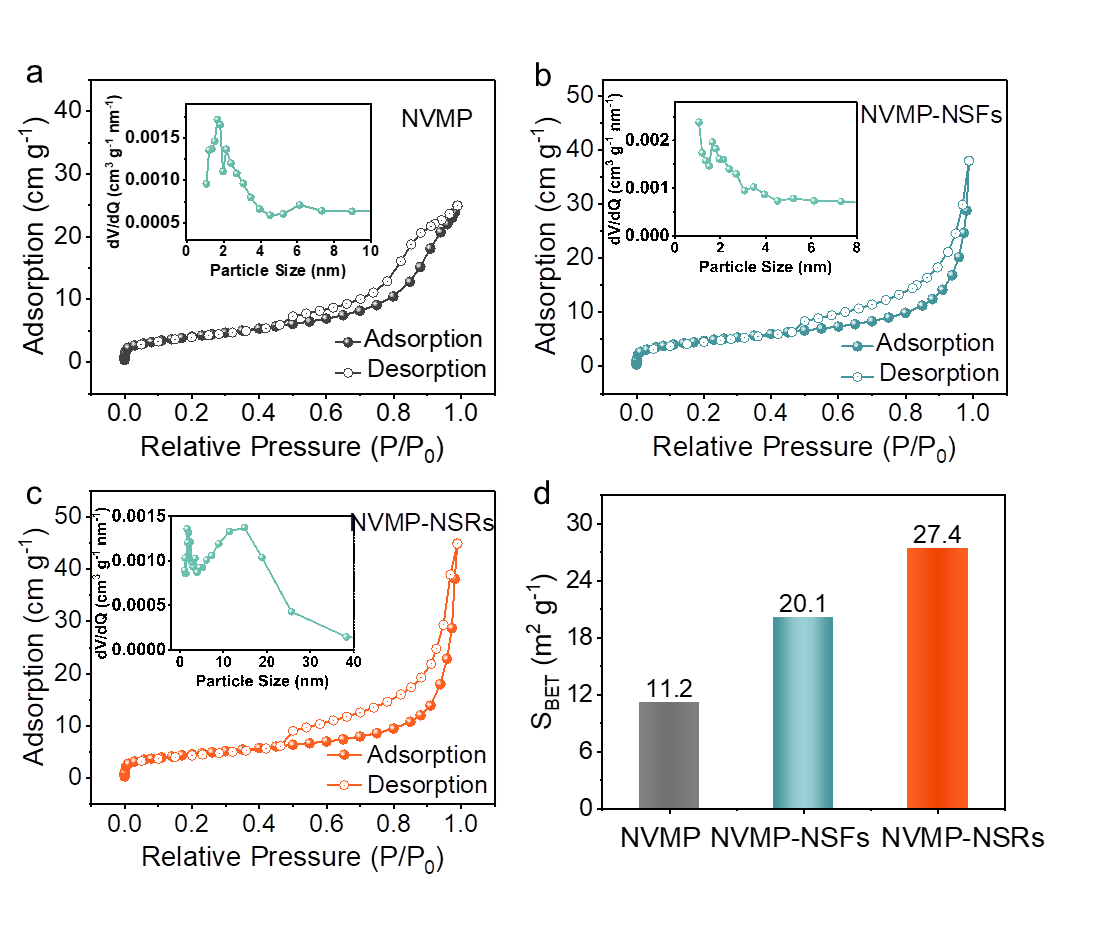


**Figure S3.** N_2_ adsorption/desorption isotherms of (a) NVMP, (b) NVMP-NSFs and (c)NVMP-NSRs; The inset shows their pore size distributions. (d) The Brunauer Emmett Teller (BET) specific areas information.


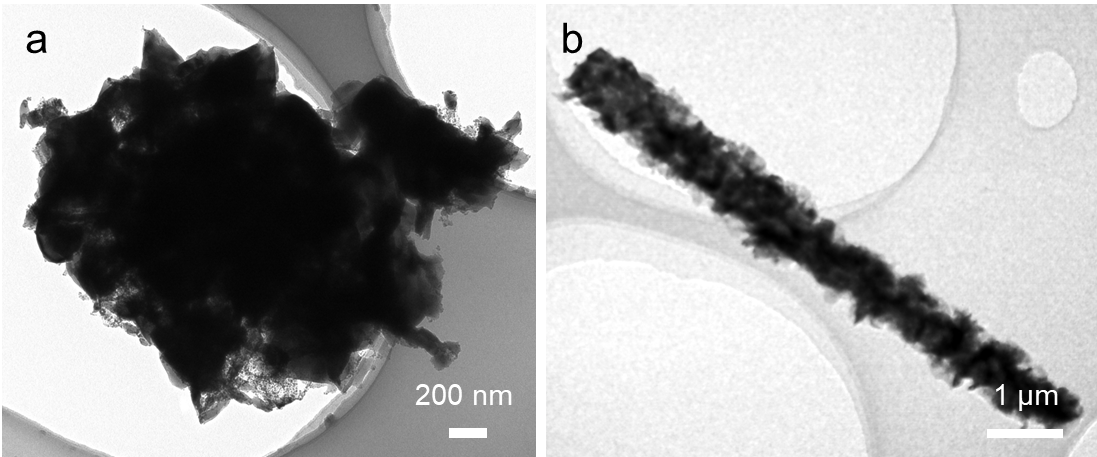


**Figure S4.** HRTEM images of NVMP-NSFs and NVMP-NSRs.

**
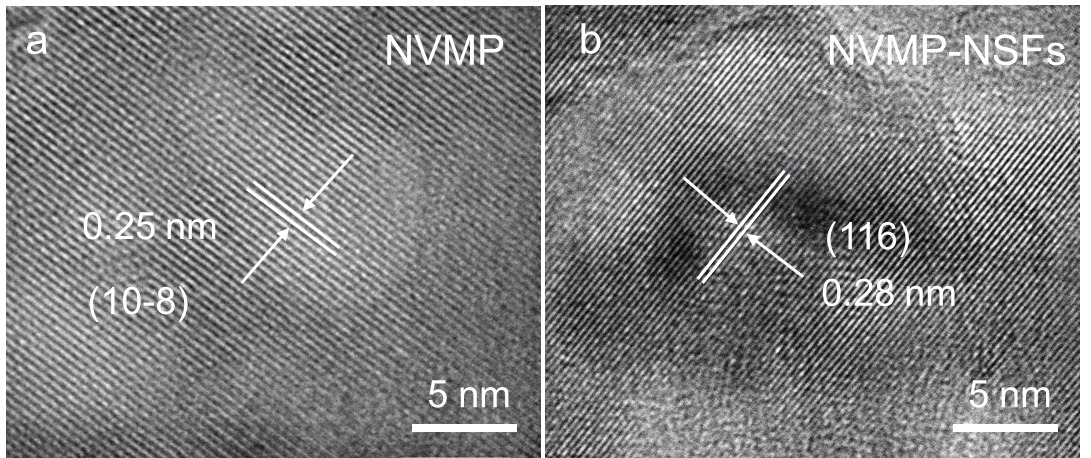
**

**Figure S5.** HRTEM images of NVMP and NVMP-NSFs.

**
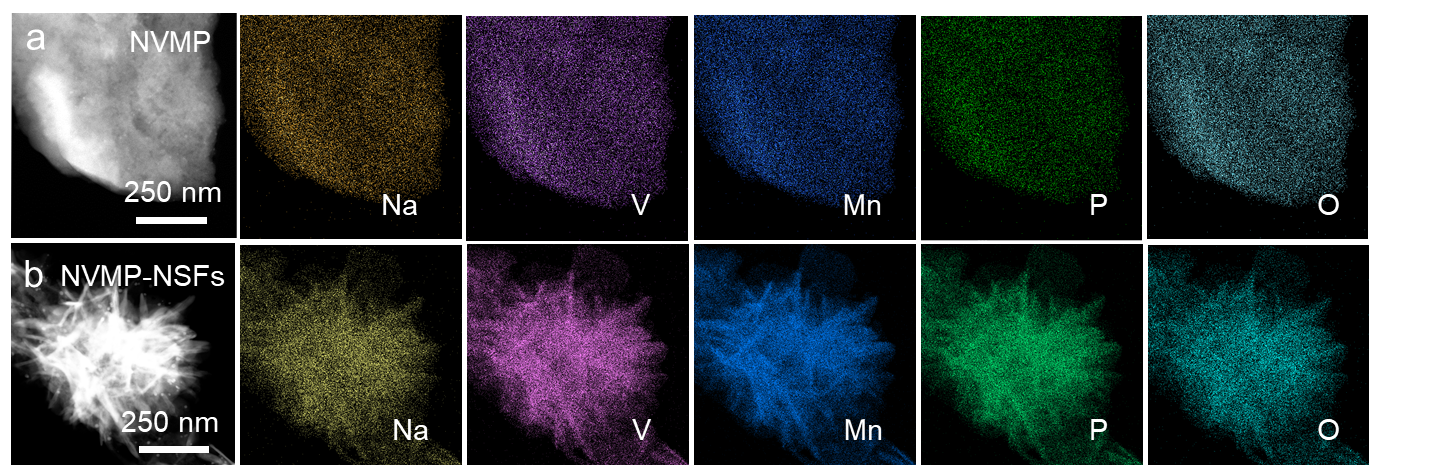
**

**Figure S6.** HRTEM image and corresponding element mapping plots of (a) NVMP and (b)NVMP-NSFs.


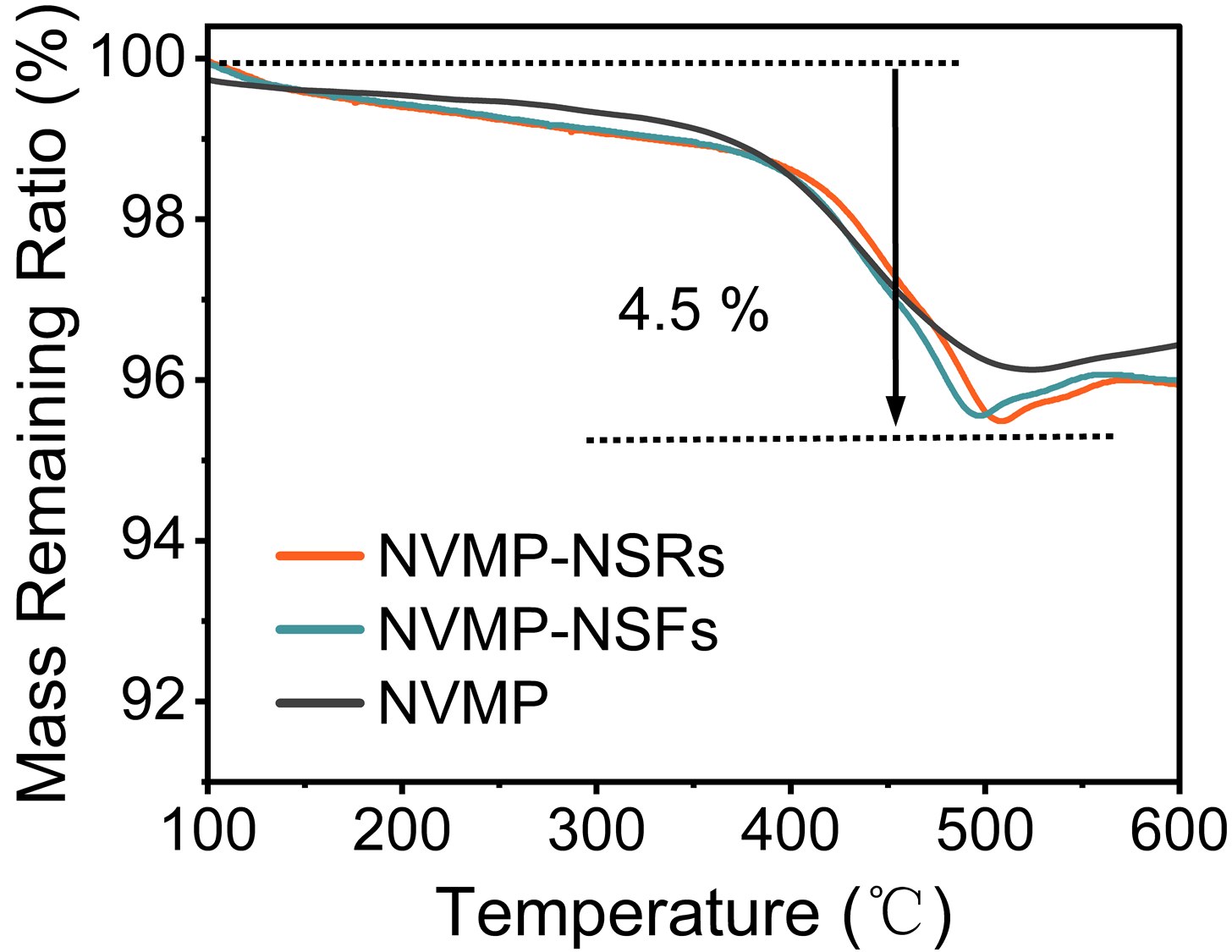


**Figure S7.** TG curves of the as-prepared samples.


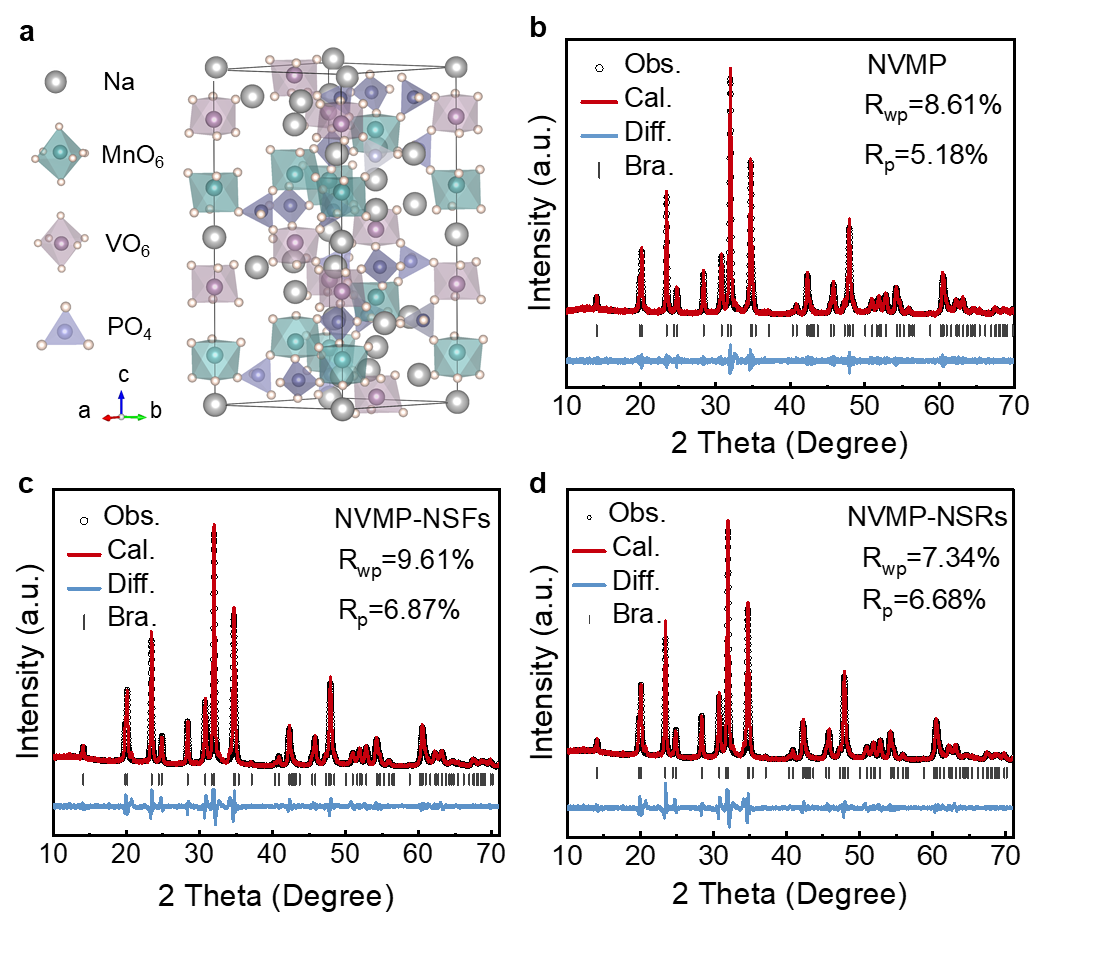


**Figure S8.** (a) Schematic diagram of the crystal structure. XRD Rietveld refinement of (b) NVMP, (c) NVMP-NSFs and (d) NVMP-NSRs.


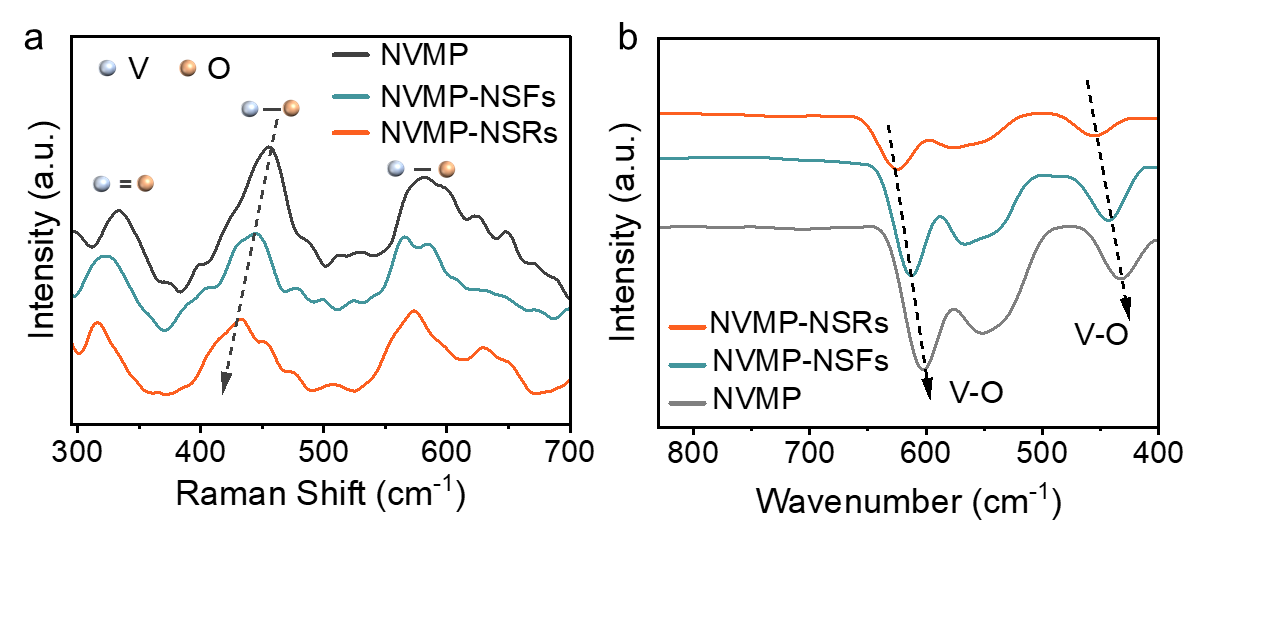


**Figure S9.** (a) Raman and (b) FTIR spectra of the as-prepared samples.


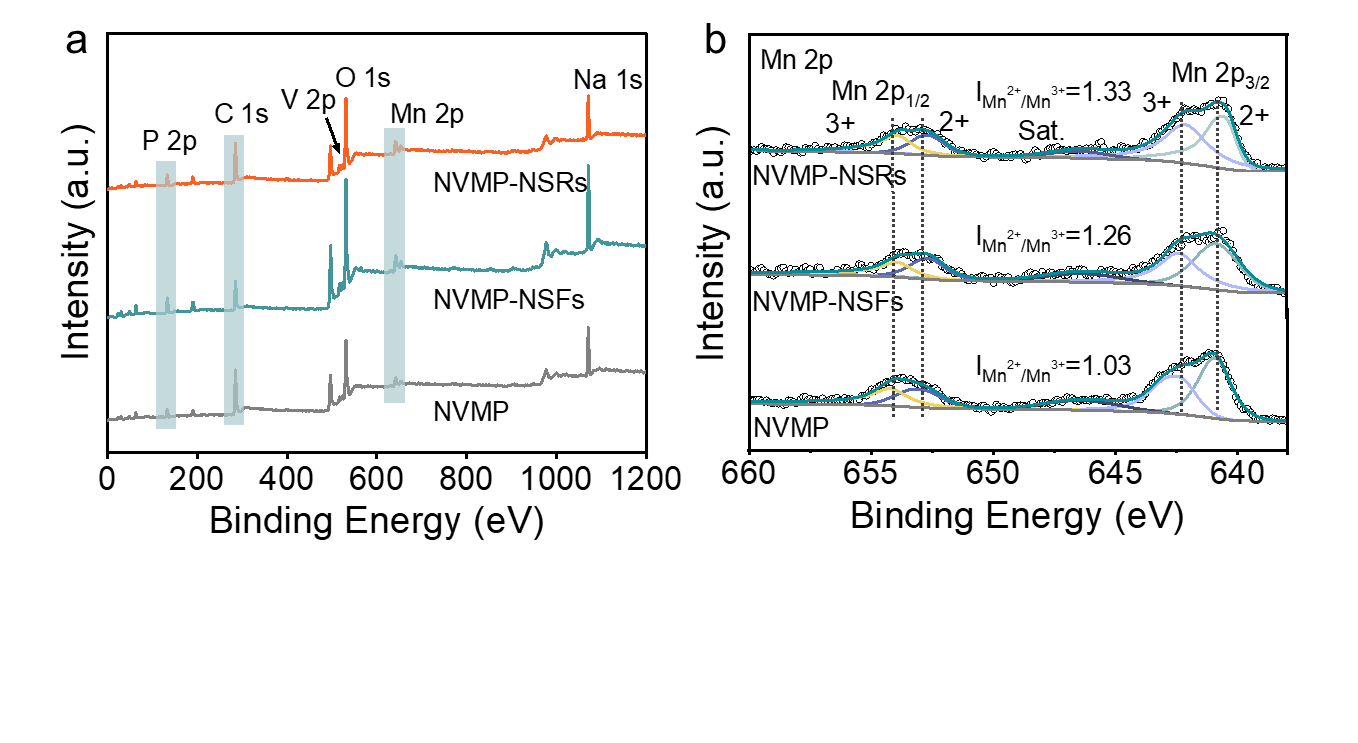


**Figure S10.** (a) XPS survey spectra with remarked element peaks, (b) high-resolution Mn 2p XPS spectra of three cathodes.


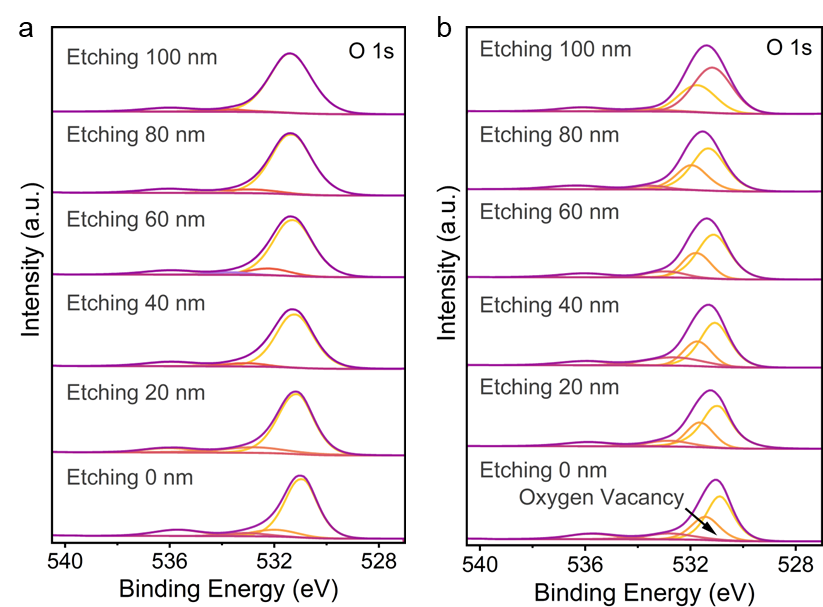


**Figure S11.** High-resolution O 1s XPS spectra of (a) NVMP and (b) NVMP-NSRs under different etching depths.

**
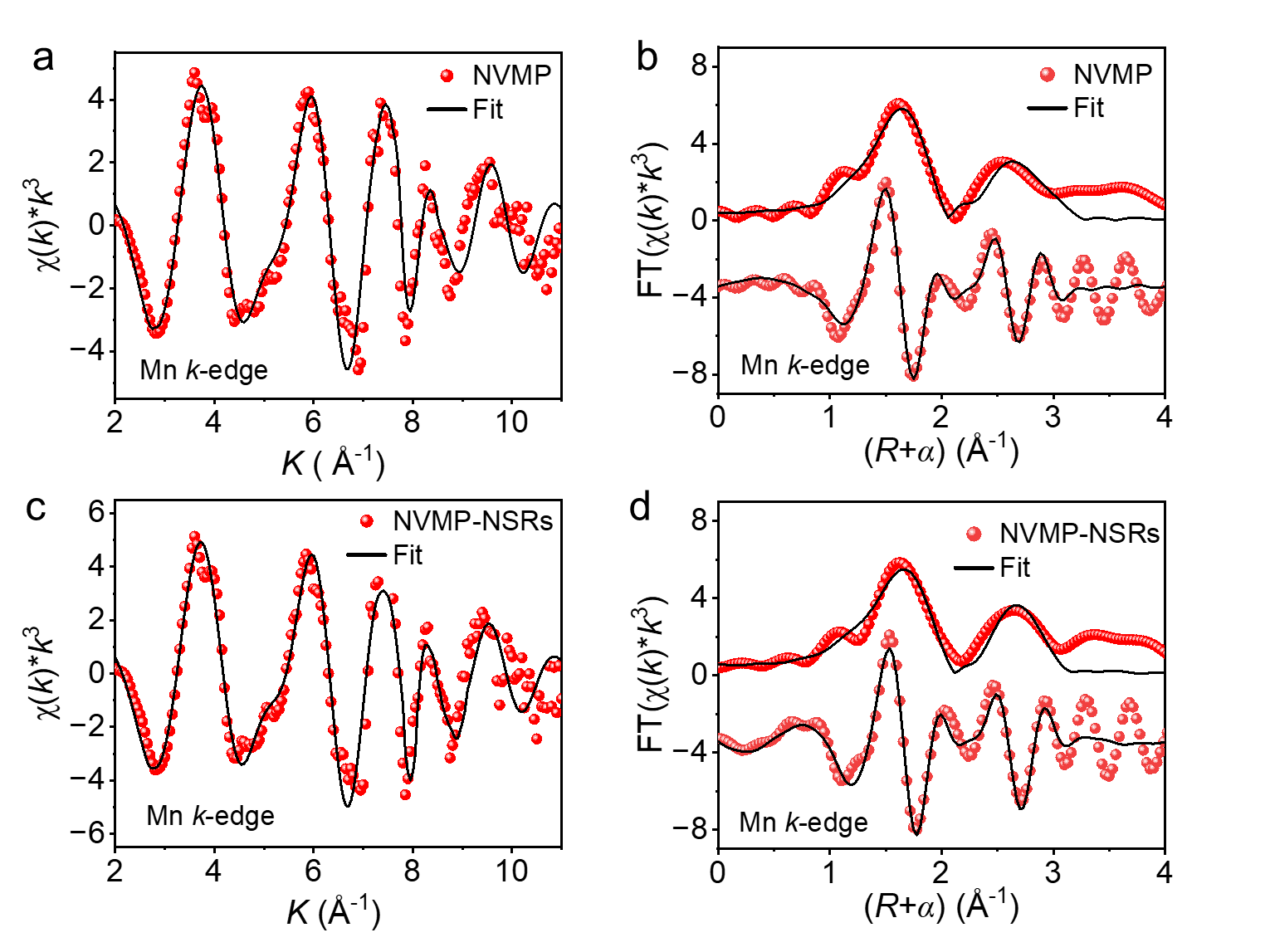
**

**Figure S12.** Mn K-edge extended X-ray absorption fine structure (EXAFS) fit in *K*-space and *R*-space for (a, b) NVMP and (c, d) NVMP-NSRs. The date is k^3^-weighted and not phase-corrected.

**
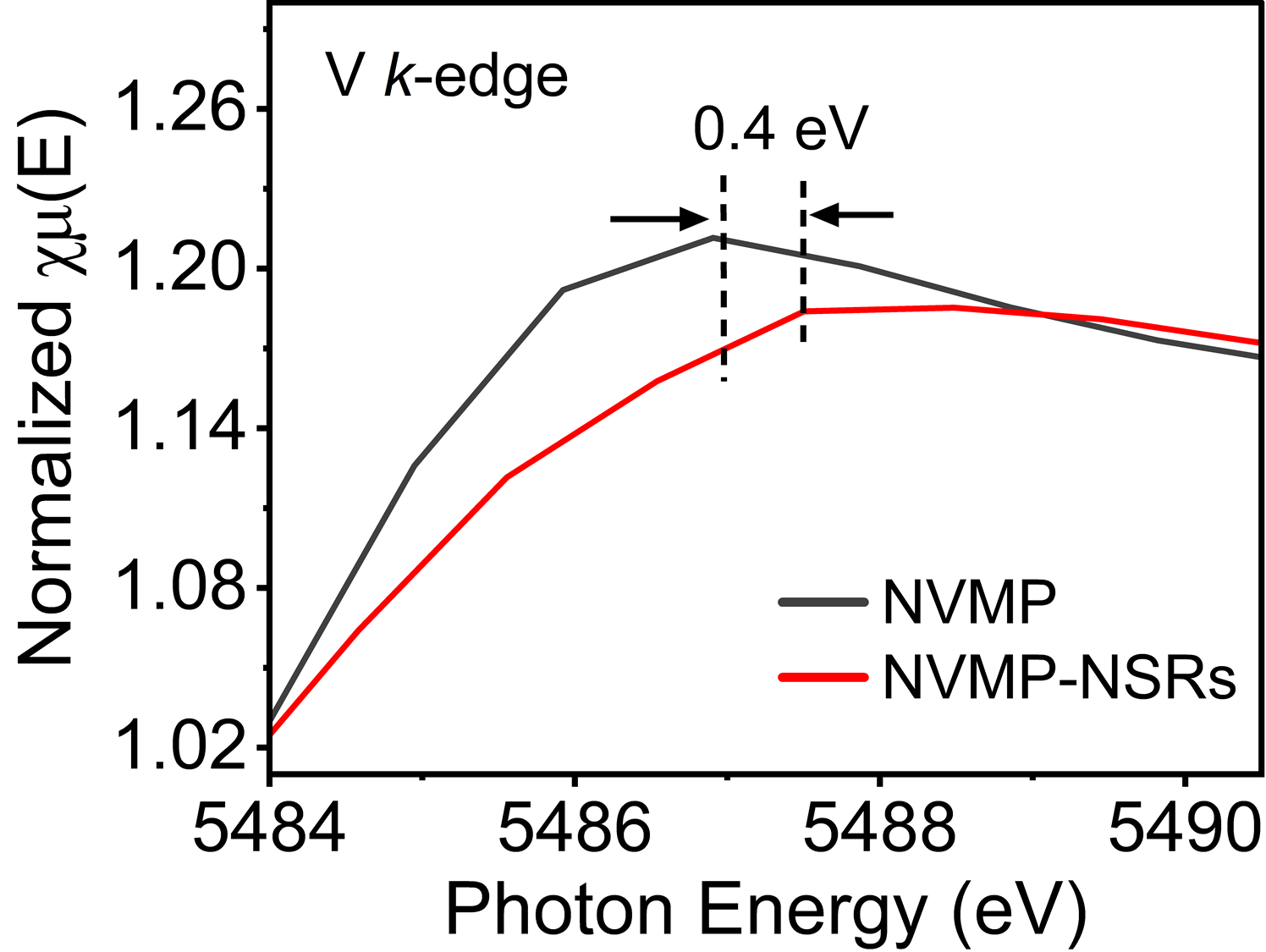
**

**Figure S13.** The enlarged V K-edge XANES for NVMP and NVMP-NSRs.


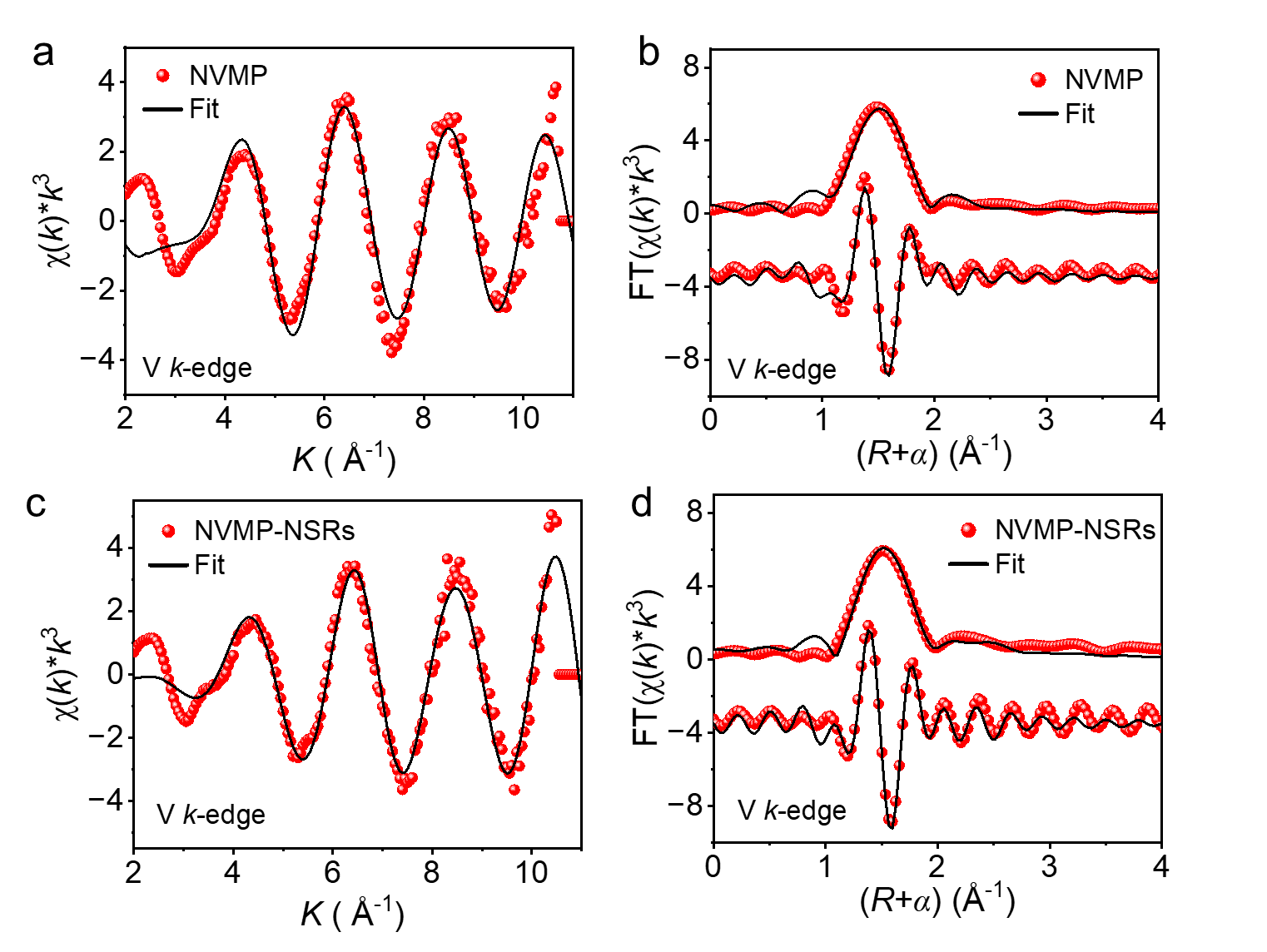


**Figure S14.** V K-edge extended X-ray absorption fine structure (EXAFS) fit in *K*-space and *R*-space for (a, b) NVMP and (c, d) NVMP-NSRs. The date is k^3^-weighted and not phase-corrected.


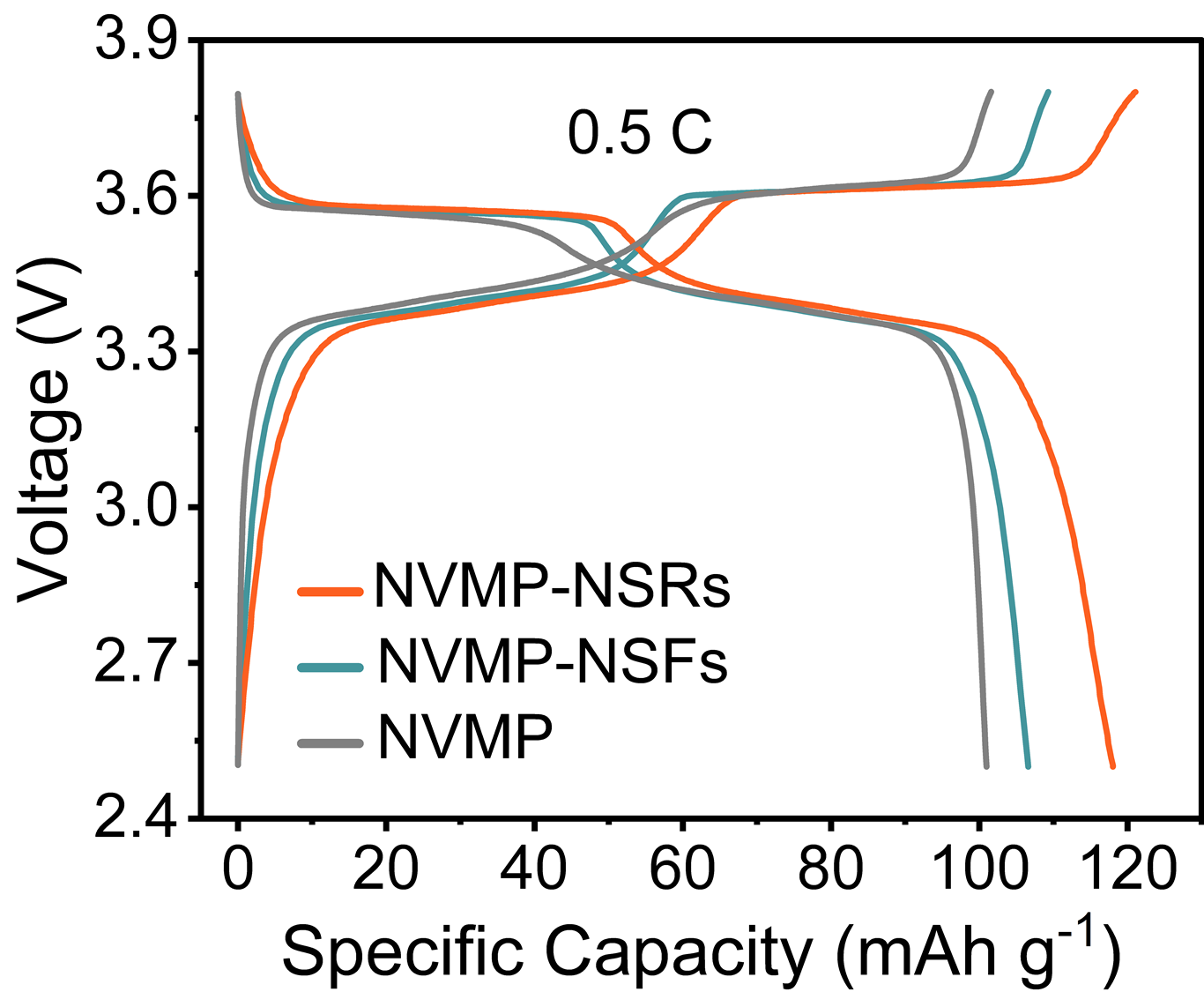


**Figure S15.** Charge/discharge curve of (a) NVMP, (b) NVMP-NSFs and (c)NVMP-NSRs.


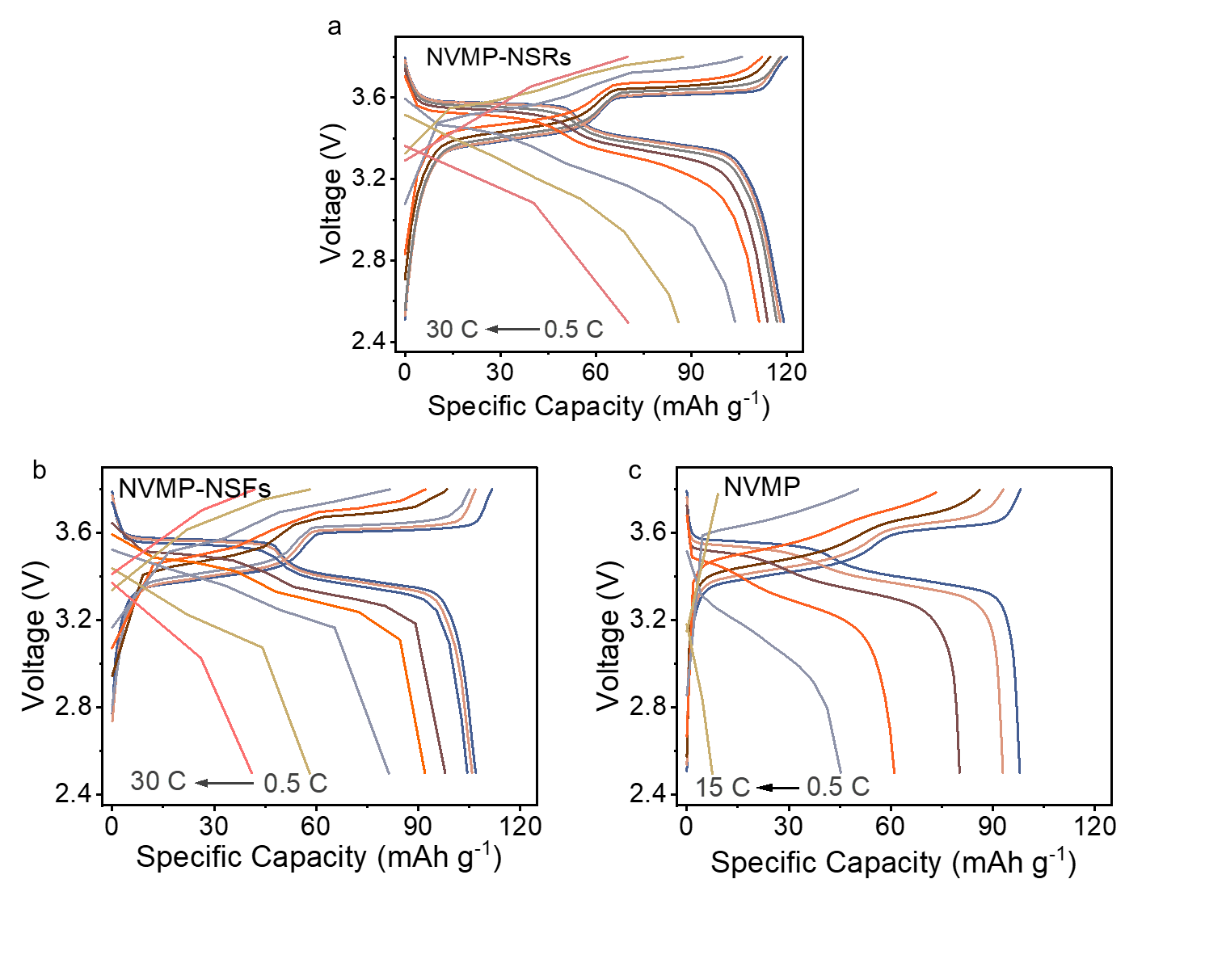


**Figure S16.** Charge/discharge curves of (a) NVMP-NSRs, (b) NVMP-NSFs, and (C) NVMP.


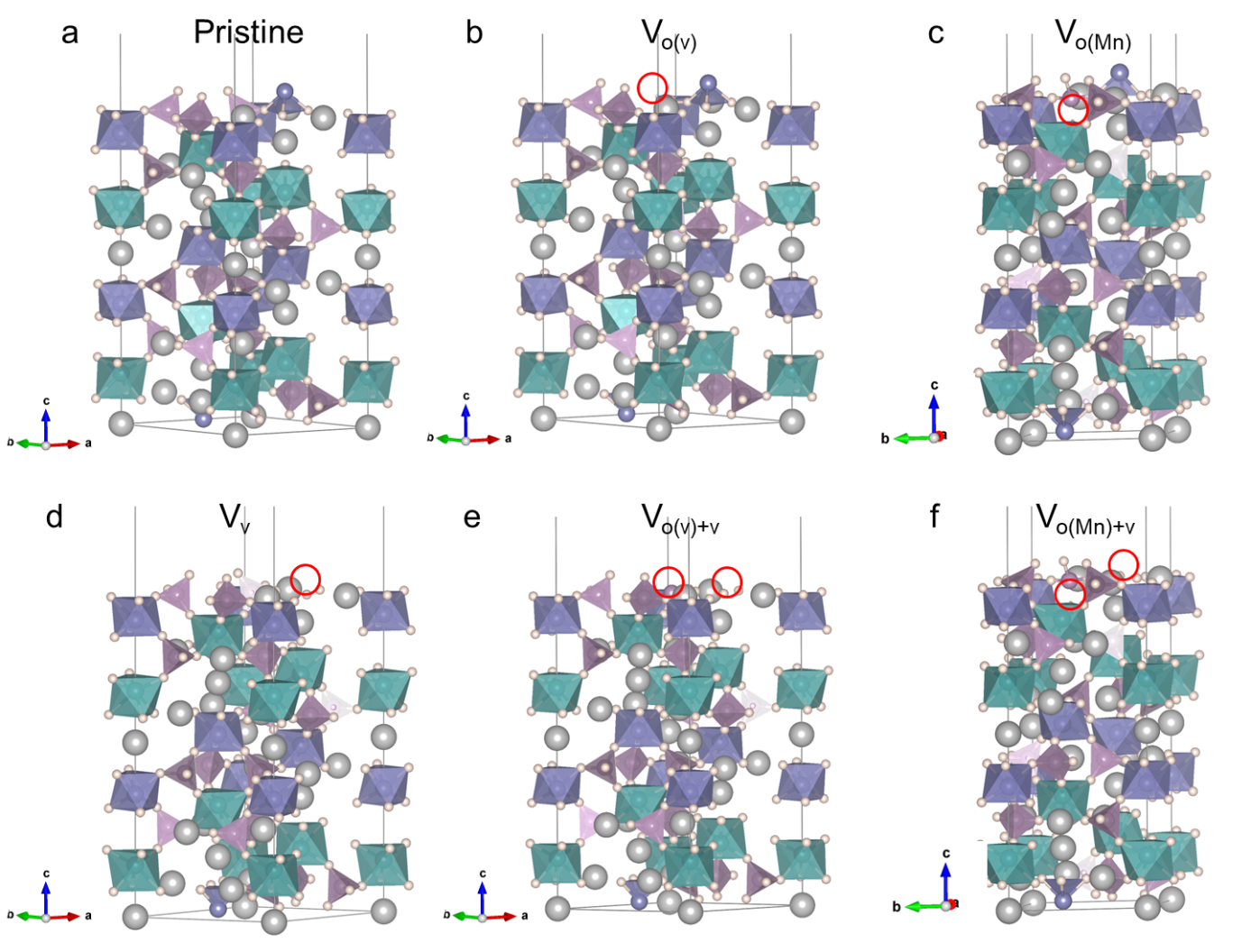


**Figure S17.** The model of intrinsic and correlated defects.


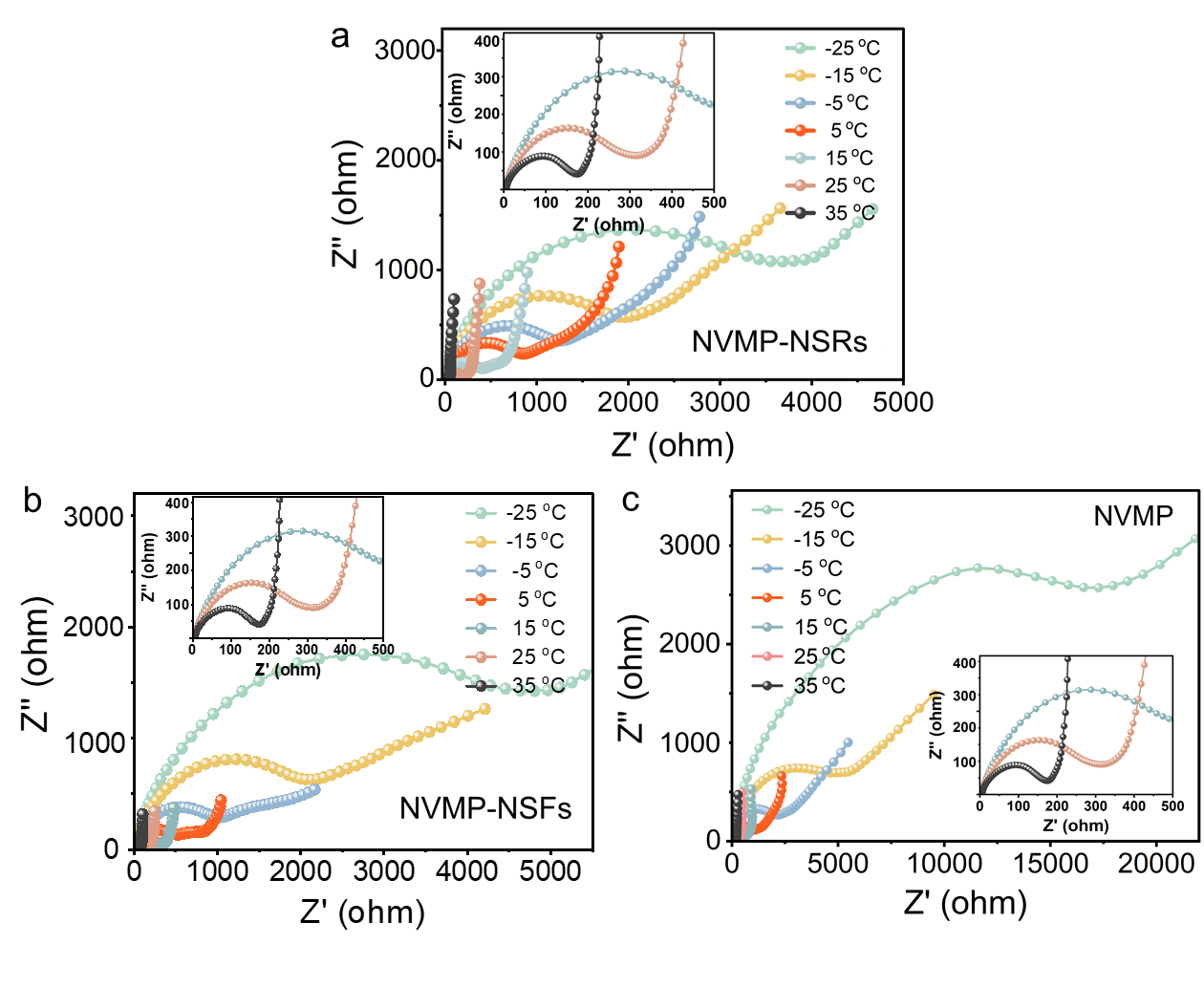


**Figure S18.** EIS spectra at different temperature of NVMP, NVMP-NSFs, and NVMP-NSRs.


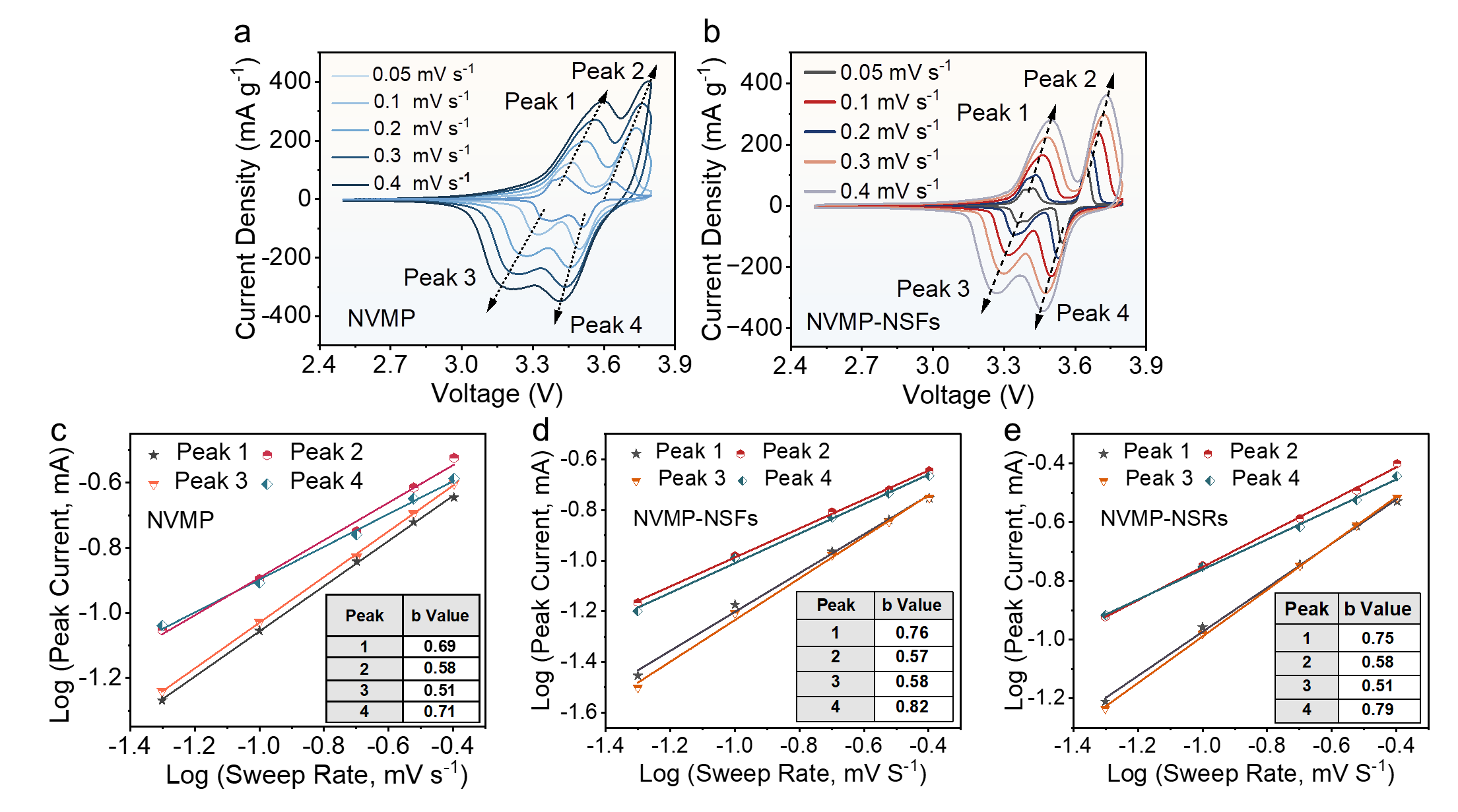


**Figure S19.** CV curves at various scan rates of (a) NVMP and (b) NVMP-NSFs. Linear fittings of log (peak current, *i*) and log (san rate, *v*) (inset: the *b* value) for (c) NVMP, (d) NVMP-NSFs and (e) NVMP-NSRs.

To further quantify the contribution of diffusion-controlled and capacitive-controlled at a specific scan rate, $i=av^{b}$is divided into two halves to form formula (S1):^[3]^

$i\left( v \right)=k_{1}v+k_{1}v^{1/2}$ (S1)

According to the above equation, the current ($i$) at a specific potential ($v$) can be divided into a capacitance limiting effect ($k_{1}v$) and a diffusion control effect ($k_{1}v^{1/2}$).


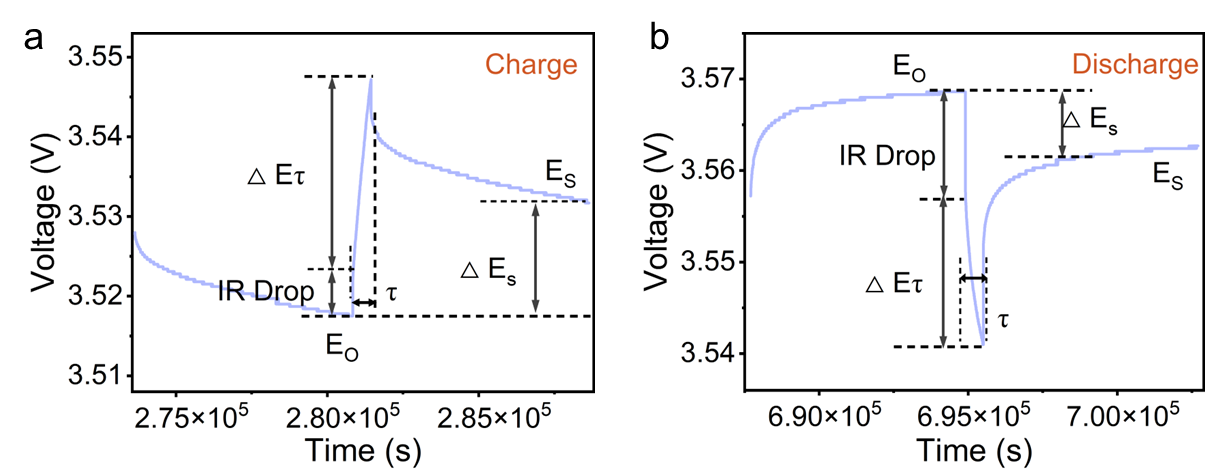


**Figure S20.** Typical profiles of single-step GITT experiment with remarked parameters during (a) charge and (b) discharge process for NVMP.

Before the GITT measurement, the cell is charged/discharged at 0.1 C for three cycles and then electrostatically charged/discharged for 40 mins, followed by 120 mins remaining to obtain an essentially steady state. The calculation of *D*_Na_^+^ is based on the equation: ^[4]^

$D_{{Na}^{+}}=4/{\pi\tau{(m_{B}V_{M}/M_{B}S)}^{2}{({\Delta E}_{S}/{\Delta E}_{\tau})}^{2} (\tau\ll l^{2}/D_{{Na}^{+}})}$ (S2)

Where $\tau$ is the duration time of the current pulse, $m_{B}$ is the mass of the active material, $M_{B}$ is the molecular weight, $V_{M}$ is the molar volume, and $S$ is the total contacting area of the electrode with electrolyte (1.13 cm^2^). ${\Delta E}_{S}$ is the difference between two consecutive stable voltages after relaxation, ${\Delta E}_{\tau}$ is the transient voltage change during a single titration step, and $l$ is the thickness of the electrode.


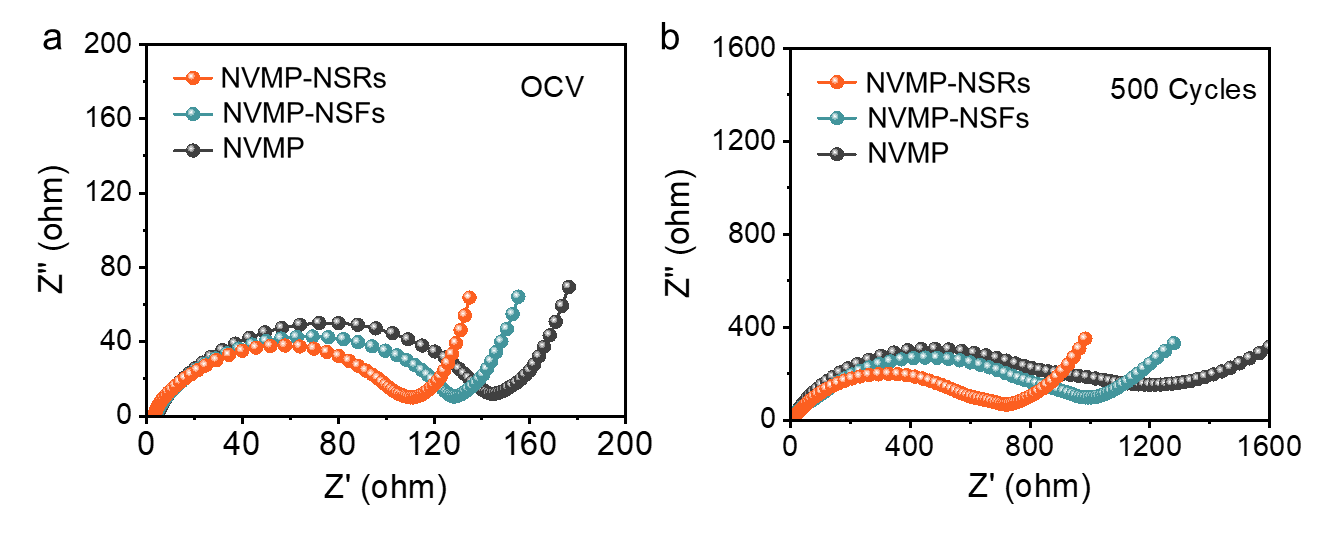


**Figure S21.** EIS plots of different samples for (a) OCV and (b) after 500 cycles at 1 C.


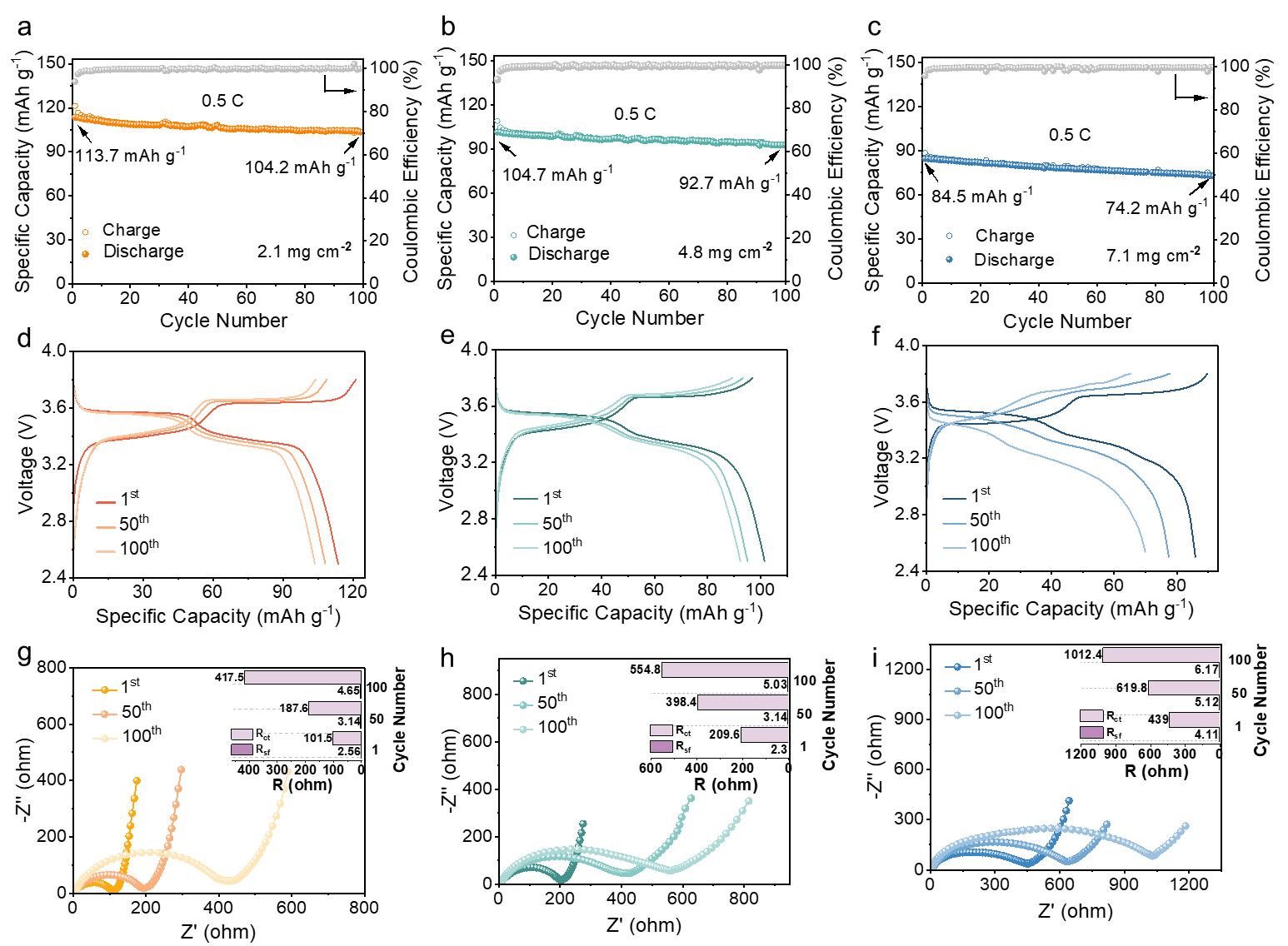


**Figure S22.** Electrochemical performance of NVMP-NSRs with different active loadings. Cycling performance with the active loadings of (a) 2.1, (b) 4.8, and (c) 7.1 mg cm^−2^ at 0.5 C, and the corresponding discharge/charge curves are given in (d), (e), and (f), respectively. Nyquist plots of NVMP-NSRs with different active loadings of (g) 2.1 (h) 4.8, and (i) 7.1 mg cm^−2^ after various cycles at 0.5 C. The insets show the corresponding fitting EIS parameters.

The cathode with loading of 2.1 mg cm^-2^ delivers high capacity of 104.2 mAh g^-1^ after 100 cycles at 0.5 C, with a capacity retention of 91.6%. When the active material loading reaches higher values of 4.8 mg cm^-2^and 7.1 mg cm^-2^, slightly decent capacity of 92.7 mAh g^-1^ and 74.2 mAh g^-1^ is observed with capacity retention of 88.5% and 87.8% after 100 cycles, respectively (**Figure S22**b and **S22**c). Note that **Figures S22**d-f provide the charge/discharge curves of 1^st^, 50^th^, and 100^th^ cycles at 0.5 C for the electrodes with active material loading of 2.1, 4.8, and 7.1 mg cm^-2^, respectively, which intuitively reflects the variation trend of their voltage polarization and reversible capacity. To reveal the electrochemical properties of NVMP-NSRs under various loading, the EIS at full-discharge state of different cycles are present in **Figures S22**g-i. Base on the RC parallel equivalent circuit provided in the inset of **Figure 4**k, the fitted kinetic parameter of NVMP-NSRs with different loading are summarized. From the inset of **Figures S22**g-i, it could be found that the values of *R*_s_ for different loading only suffer from imperceptible increase upon cycling, indicating the negligible contribution of *R*_s_ to the performance evolutions. In contrast, the *R*_sf_ values undergo a gentle rise in the subsequent cycles, which lead to the decay of reversible capability under high loading, this mainly ascribed to the deterioration of ionic and electronic conductivity within the test electrode induced by the consumption or lack of electrolyte.


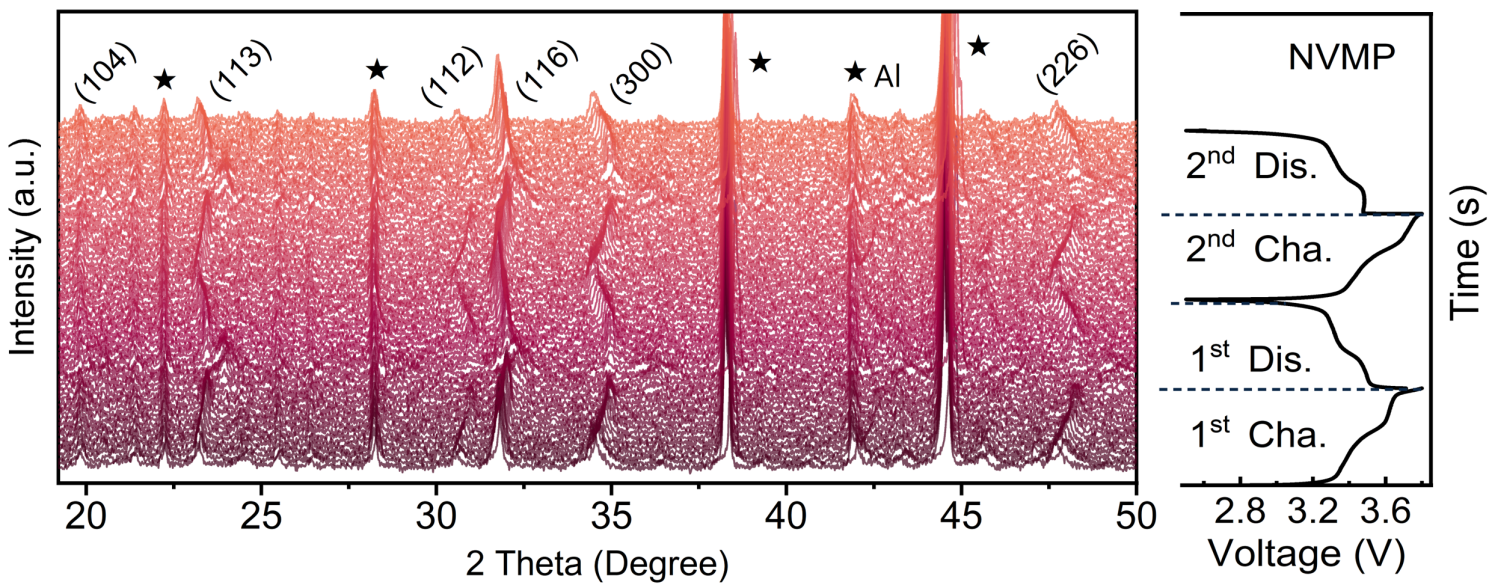


**Figure S23.** *In situ* XRD patterns of NVMP during the first two cycles at 0.2 C.


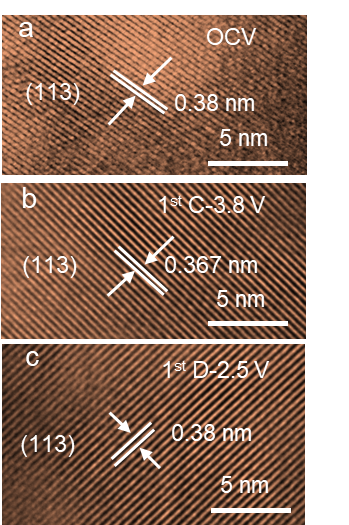


**Figure S24.** HRTEM images of NVMP-NSRs at different states of (a) OCV, (b) charged to 3.8 V (C-3.8 V) and (c) discharged to 2.5 V (D-2.5 V).


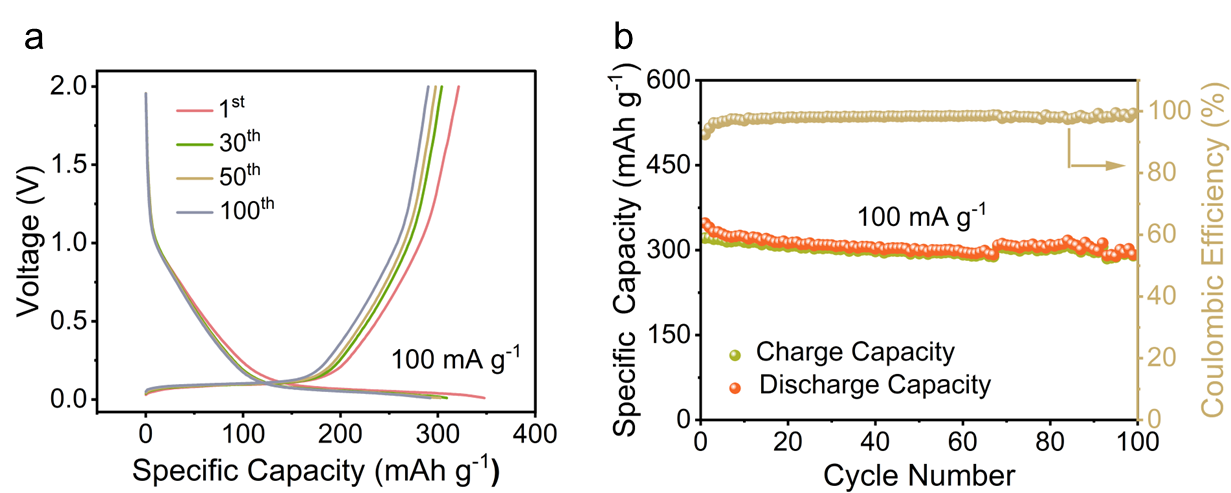


**Figure S25.** (a) Galvanostatic charge/discharge profiles and (b) cycling performance of Hard carbon at 100 mA g^-1^ in the potential range of 0.01-2.0 V *vs.* Na^+^/Na.

**Table S1.** XRD refinement data of NVMP.

| **NVMP**  *a* = *b* = 8.8116 Å *c* = 21.5936 Å *V* = 1492.54 Å^3^  α = β = 90° γ = 120° R-3c  *R*_p_ = 8.61% *R*_wp_ = 5.18% *R*_exp_ = 6.16% *X*^2^ = 1.754 | | | | | |
| --- | --- | --- | --- | --- | --- |
| Atom | Np | *x* | *y* | *z* | Occ. |
| Na1 | 6b | 0.0000 | 0.0000 | 0.0000 | 1.0219 |
| Na2 | 18e | 0.6429 | 0.0000 | 0.2500 | 0.9697 |
| V | 12c | 0.0000 | 0.0000 | 0.1486 | 0.5000 |
| Mn | 12c | 0.0000 | 0.0000 | 0.1486 | 0.5000 |
| O1 | 36f | 0.1890 | 0.1678 | 0.0889 | 1.000 |
| O2 | 36f | 0.0218 | 0.2074 | 0.1961 | 1.000 |
| P | 18e | 0.2918 | 0.0000 | 0.2500 | 1.000 |

**Table S2.** XRD refinement data of NVMP-NSFs.

| **NVMP-NSFs**  *a* = *b* = 8.7965 Å *c* = 21.6247 Å *V* = 1489.96 Å^3^  α = β = 90° γ = 120° R-3c  *R*_p_ = 9.61% *R*_wp_ = 6.87% *R*_exp_ = 5.16% *X*^2^ = 1.654 | | | | | |
| --- | --- | --- | --- | --- | --- |
| Atom | Np | *x* | *y* | *z* | Occ. |
| Na1 | 6b | 0.0000 | 0.0000 | 0.0000 | 1.0171 |
| Na2 | 18e | 0.6301 | 0.0000 | 0.2500 | 0.9799 |
| V | 12c | 0.0000 | 0.0000 | 0.1485 | 0.5000 |
| Mn | 12c | 0.0000 | 0.0000 | 0.1485 | 0.5000 |
| O1 | 36f | 0.1916 | 0.1673 | 0.0886 | 1.000 |
| O2 | 36f | 0.0311 | 0.2146 | 0.1934 | 1.000 |
| P | 18e | 0.2906 | 0.0000 | 0.2500 | 1.000 |

**Table S3.** XRD refinement data of NVP-NRs.

| **NVMP-NSRs**  *a* = *b* = 8.7817 Å *c* = 21.8047 Å *V* = 1488.94 Å  α = β = 90° γ = 120° R-3c  *R*_p_ = 7.34% *R*_wp_ = 6.88% *R*_exp_ = 5.33% *X*^2^ = 1.854 | | | | | |
| --- | --- | --- | --- | --- | --- |
| Atom | Np | *x* | *y* | *z* | Occ. |
| Na1 | 6b | 0.0000 | 0.0000 | 0.0000 | 1.0003 |
| Na2 | 18e | 0.6368 | 0.0000 | 0.2500 | 0.9812 |
| V | 12c | 0.0000 | 0.0000 | 0.1483 | 0.5000 |
| Mn | 12c | 0.0000 | 0.0000 | 0.1483 | 0.5000 |
| O1 | 36f | 0.1897 | 0.1804 | 0.0846 | 1.000 |
| O2 | 36f | 0.0198 | 0.2108 | 0.1943 | 1.000 |
| P | 18e | 0.2976 | 0.0000 | 0.2500 | 1.000 |

**Table S4.** *K*-edge EXAFS curve fitting parameters of NVMP and NVMP-NSRs.

| Sample | Shell | *N* | *R* (Å) | σ^2^ (Å^2^) | *R* factor |
| --- | --- | --- | --- | --- | --- |
| NVMP | Mn-O | 4.27 | 1.99 | 0.003 | 0.002 |
|  | V-O | 5.91 | 1.79 | 0.004 | 0.003 |
| NVMP-NSRs | Mn-O | 4.24 | 1.89 | 0.004 | 0.005 |
|  | V-O | 4.27 | 1.51 | 0.002 | 0.002 |

*N*: coordination number.

*R*: interatomic distance between central atoms and backscatter atoms.

σ^2^: Debye-Waller factor to characterize both thermal and structural disorders.

*R* factor: the indicator for the goodness of the fit.

Fitting range: NVMP (3 ≤ k ≤ 11.5, 1.0 ≤ R ≤ 3.0); NVMP-NSRs (3 ≤ k ≤ 12.0, 1.0 ≤ R ≤ 3.0).

**Table S5.** The electrochemical performance comparison between NVMP-NSRs and other reported phosphate cathodes.

| Cathode | Voltage window (V) | Capacity | Rate performance | Cycling stability |
| --- | --- | --- | --- | --- |
| Na_4_FeV(PO_4_)_3_@C^[5]^ | 1.8-4.3 | 99.9 mAh g^–1^ at 0.1 C  (1 C = 175.6 mA g^−1^) | 92.4 mAh g^–1^ at 0.1 C  86.7 mAh g^–1^ at 0.2 C  79.3 mAh g^–1^ at 0.5 C  73.1 mAh g^–1^ at 1 C  65.2 mAh g^–1^ at 2 C  51.2 mAh g^–1^ at 5 C  34.7 mAh g^–1^ at 10 C | 96.3% capacity retention at 0.1 C after 100 cycles. |
| Na_4_MnV(PO_4_)_3_@C^[6]^ | 2.5-3.8 | 90.1 mAh g^–1^ at 1 C  (1 C = 110.0 mA g^−1^) | 97.8 mAh g^–1^ at 0.2 C  95.6 mAh g^–1^ at 0.5 C  93.0 mAh g^–1^ at 1 C  89.5 mAh g^–1^ at 2 C  82.2 mAh g^–1^ at 5 C  60.0 mAh g^–1^ at 10 C | 91.2% capacity retention at 1 C after 150 cycles. |
| Na_3.95_MnV_0.95_Zr_0.05_(PO_4_)_3_/C^[7]^ | 2.5–3.8 | 100.2 mAh g^–1^ at 0.2 C  (1 C = 110.0 mA g^−1^) | 95.4 mAh g^–1^ at 0.2 C  92.1 mAh g^–1^ at 0.5 C  88.2 mAh g^–1^ at 1 C  84.0 mAh g^–1^ at 2 C  81.1 mAh g^–1^ at 5 C  77.9 mAh g^–1^ at 10 C  75.3 mAh g^–1^ at 20 C | 83.1% capacity retention at 5 C after 1000 cycles. |
| Na_3_V_2_(PO_4_)_2_O_2_F-Cl^[8]^ | 2.5–4.5 | 118.1 mAh g^–1^ at 0.2 C  (1 C = 117.8 mA g^−1^) | 118.0 mAh g^–1^ at 0.2 C  115.1 mAh g^–1^ at 0.5 C  110.2 mAh g^–1^ at 1 C  105.5 mAh g^–1^ at 2 C  100.1 mAh g^–1^ at 5 C  92.3 mAh g^–1^ at 10 C  73.1 mAh g^–1^ at 20 C  60.9 mAh g^–1^ at 30 C | 98.6% capacity retention at 1 C after 200 cycles.  82.3% capacity retention at 10 C after 1000 cycles. |
| HE-NASICON^[9]^ | 1.5–4.5 | 161.3 mAh g^–1^ at 0.1 C  (1 C = 150.0 mA g^−1^) | 163.0 mAh g^–1^ at 0.1 C  138.3 mAh g^–1^ at 0.2 C  116.2 mAh g^–1^ at 0.5 C  106.4 mAh g^–1^ at 1 C  99.4 mAh g^–1^ at 2 C  87.7 mAh g^–1^ at 5 C  76.1 mAh g^–1^ at 10 C  58.8 mAh g^–1^ at 20 C | 91.2% capacity retention at 0.5 C after 100 cycles. 85.3% capacity retention at 5 C after 1000 cycles. |
| Na_3_V_1.6_Cr_0.4_(PO_4_)_3_^[10]^ | 2.5–4.0 | 100.9 mAh g^-1^ at 0.1 C  (1 C = 117.8 mA g^−1^) | 100 mAh g^–1^ at 0.1 C  95.2 mAh g^–1^ at 0.2 C  91.5 mAh g^–1^ at 0.5 C  90.5 mAh g^–1^ at 1 C  89.1 mAh g^–1^ at 2 C  88.0 mAh g^–1^ at 5 C  81.2 mAh g^–1^ at 10 C  80.3 mAh g^–1^ at 20 C | 91.1% capacity retention at 1 C after 100 cycles. |
| Na_2.5_VTi_0.5_Al_0.5_(PO_4_)_3_^[11]^ | 1.5–4.3 | 94 mAh g^-1^ at 0.1 C  (1 C = 117 mA g^−1^) | 93 mAh g^–1^ at 0.1 C  79 mAh g^–1^ at 0.2 C  67 mAh g^–1^ at 0.5 C  57 mAh g^–1^ at 1 C | 50% capacity retention at 0.2 C after 100 cycles. |
| Na_4_MnV(PO_4_)_3_@C^[12]^ | 2.5–3.8 | 90 mAh g^-1^ at 1 C  (1 C = 117 mA g^−1^) | 97 mAh g^–1^ at 0.2 C  95 mAh g^–1^ at 0.5 C  93 mAh g^–1^ at 1 C  89 mAh g^–1^ at 2 C  82 mAh g^–1^ at 5 C  60 mAh g^–1^ at 10 C | 99% capacity retention at 0.2 C after 60 cycles.  80% capacity retention at 5 C after 800 cycles. |
| Na_3_V_2_(PO_4_)_3_-Na_3_Fe_2_(PO_4_) (P_2_O_7_)^[13]^ | 2.0–4.3 | 113 mAh g^-1^ at 0.1 C  (1 C = 118 mA g^−1^) | 110 mAh g^–1^ at 0.1 C  105 mAh g^–1^ at 0.2 C  100 mAh g^–1^ at 0.5 C  97 mAh g^–1^ at 1 C  90 mAh g^–1^ at 2 C  80 mAh g^–1^ at 5 C  70 mAh g^–1^ at 10 C  60 mAh g^–1^ at 15 C | 78.6% capacity retention at 0.5 C after 500 cycles. |
| Na_4_Mn_0.9_CrMg_0.1_(PO_4_)_3_/C^[14]^ | 1.4–4.6 | 150 mAh g^-1^ at 0.1 C  (1 C = 156 mA g^−1^) | 154 mAh g^–1^ at 0.1 C  149 mAh g^–1^ at 0.2 C  131 mAh g^–1^ at 0.5 C  121 mAh g^–1^ at 1 C  107 mAh g^–1^ at 2 C  92 mAh g^–1^ at 5 C  79 mAh g^–1^ at 10 C  70 mAh g^–1^ at 15 C | 90.4% capacity retention at 2 C after 250 cycles.  92.7% capacity retention at 5 C after 500 cycles. |
| **This Work** | 2.5–3.8 | 120.1 mAh g^-1^ at 0.5 C  (1 C = 110 mA g^−1^) | 120.1 mAh g^–1^ at 0.5 C  118.9 mAh g^–1^ at 1 C  117.5 mAh g^–1^ at 2 C  115.1 mAh g^–1^ at 5 C  111.4 mAh g^–1^ at 10 C  103.9 mAh g^–1^ at 15 C  85.5 mAh g^–1^ at 20 C  70.9 mAh g^–1^ at 30 C | 91.1 % capacity retention at 5 C after 500 cycles.  94.5 % capacity retention at 20 C after 3000 cycles. |

**References**

1. Blöchl, Peter E, *Phys. Rev. B* 1994, **50**, 17953.
2. a) J.P. Perdew, K. Burke, M. Ernzerhof, *Phys. Rev. Lett*. 1996, **77**, 3865; b) G. Henkelman, B. P. Uberuaga, H. Jónsson, *J. Chem. Phys* 2000, **113**, 9901-9904.
3. S. Li, X. Xu, W. Chen, J. Zhao, K. Wang, J. Shen, X. Chen, X. Lu, X. Jiao, Y. Liu, Y. Bai, *Energy Storage Mater.* 2024, **65**, 103108.
4. B. Patra, R. Hegde, A. Natarajan, D. Deb, D. Sachdeva, N. Ravishankar, K. Kumar, G. S. Gautam, P. Senguttuvan, *Adv. Energy Mater.* 2024**,** **14**, 2304091.
5. F. Lu, J. Wang, S. Chang, L. He, M. Tang, Q. Wei, S. Mo, X. Kuang, *Carbon* 2022, **196**, 562-572.
6. K. Wang, X. Huang, C. C. Luo, Z. Zhang, H. Wang, T. Zhou, *ACS Appl. Energy Mater.* 2022, **5**, 15701-15709.
7. Z. Z. Wang, G. J. Cui, Q. F. Zheng, X. Y. Ren, Q. H. Yang, S. Q. Yuan, X. Bao, C. J. Shu, Y. X. Zhang, L. S. Li, Y. S. He, L. W. Chen, Z. F. Ma, X. Z. Liao, *Small* 2023, **19**, 2206987.
8. J. J. Wang, J. Z. Kang, Z. Y. Gu, Q. H. Liang, X. Y. Zhao, X. M. Wang, R. S. Guo, H. Yu, C. F. Du, X. L. Wu, *Adv. Funct. Mater.* 2022, **32**, 2109694.
9. Z. Y. Gu, J. Z. Guo, J. M. Cao, X. T. Wang, X. X. Zhao, X. Y. Zheng, W. H. Li, Z. H. Sun, H. J. Liang, X. L. Wu, *Adv. Mater.* 2022, **34**, 2110108.
10. P. Lavela, R. Klee, J. L. Tirado, *J. Power Sources* 2021, **495**, 229811.
11. Z. C. Li, C. Sun, M. Li, X. Y. Wang, Y. Li, X. Y. Yuan, H. B. Jin, Y. J. Zhao. *Adv. Funct. Mater.* 2024, **11**, 2315114.
12. P. Hu, T. Zhu, C. Cai, B. Mai, C. Yang, J. Ma, L. Zhou, H. J. Fan, L. Mai, *Adv. Funct. Mater.* 2022, **32**, 2208051.
13. A. Zhao, T. Yuan, P. Li, C. Liu, H. Cong, X. Pu, Z. Chen, X. Ai, H. Yang, Y. Cao, *Nano Energy* 2022, **91**, 106680.
14. J. Li, X. Zhao, P. He, Y. Liu, Y, Wang, S, Li, X, Qu, Y, Liu, L, Jiao, *Small* 2022, **18**, 2202879.
